# Advanced Immunolabeling Method for Optical Volumetric Imaging Reveals Dystrophic Neurites of Dopaminergic Neurons in Alzheimer’s Disease Mouse Brain

**DOI:** 10.1007/s12035-023-03823-9

**Published:** 2023-12-04

**Authors:** Soonbong Baek, Jaemyung Jang, Hyun Jin Jung, Hyeyoung Lee, Youngshik Choe

**Affiliations:** 1 Developmental Disorders & Rare Diseases Research Group, Korea Brain Research Institute, 61 Cheomdan-ro, Daegu, 41062 Republic of Korea; 2 Division of Applied Bioengineering, Dong-eui University, Busanjin-gu, Busan, 47340 Republic of Korea

**Keywords:** Neural circuits, Volumetric imaging, Alzheimer’s disease, Dopamine neurons, Axon dystrophy

## Abstract

Optical brain clearing combined with immunolabeling is valuable for analyzing molecular tissue structures, including complex synaptic connectivity. However, the presence of aberrant lipid deposition due to aging and brain disorders poses a challenge for achieving antibody penetration throughout the entire brain volume. Herein, we present an efficient brain-wide immunolabeling method, the immuno-active clearing technique (iACT). The treatment of brain tissues with a zwitterionic detergent, specifically SB3-12, significantly enhanced tissue permeability by effectively mitigating lipid barriers. Notably, Quadrol treatment further refines the methodology by effectively eliminating residual detergents from cleared brain tissues, subsequently amplifying volumetric fluorescence signals. Employing iACT, we uncover disrupted axonal projections within the mesolimbic dopaminergic (DA) circuits in 5xFAD mice. Subsequent characterization of DA neural circuits in 5xFAD mice revealed proximal axonal swelling and misrouting of distal axonal compartments in proximity to amyloid-beta plaques. Importantly, these structural anomalies in DA axons correlate with a marked reduction in DA release within the nucleus accumbens. Collectively, our findings highlight the efficacy of optical volumetric imaging with iACT in resolving intricate structural alterations in deep brain neural circuits. Furthermore, we unveil the compromised integrity of DA pathways, contributing to the underlying neuropathology of Alzheimer’s disease. The iACT technique thus holds significant promise as a valuable asset for advancing our understanding of complex neurodegenerative disorders and may pave the way for targeted therapeutic interventions.

**Graphical Abstract:**

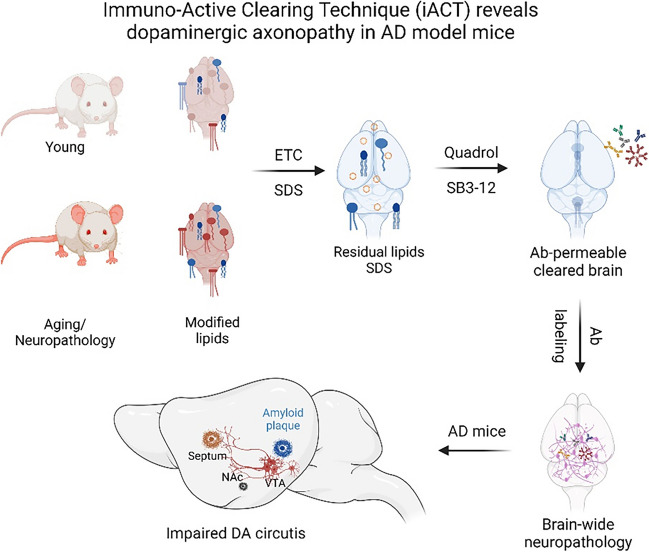

The axonal projection of DA neurons in the septum and the NAc showed dystrophic phenotypes such as growth cone-like enlargement of the axonal terminus and aggregated neurites. Brain-wide imaging of structural defects in the neural circuits was facilitated with brain clearing and antibody penetration assisted with SB3-12 and Quadrol pre-treatment. The whole volumetric imaging process could be completed in a week with the robust iACT method. Created with https://www.biorender.com/.

**Supplementary Information:**

The online version contains supplementary material available at 10.1007/s12035-023-03823-9.

## Introduction

Neuronal circuits are organized across multiple spatiotemporal scales and form highly complex networks with synaptic interconnections, which makes it challenging to study the structure-function relationship of the brain [1, 2]. Volumetric optical clearing provides valuable approaches for analyzing sophisticated synaptic connectivity or neuronal networks in the nervous system [3, 4]. Recent advancements in light sheet fluorescence imaging combined with three-dimensional (3D) rendering in optically cleared tissue samples have successfully visualized unknown brain-wide phenotypes, including cellular distribution, circuitry, and specific marker expression [5–8].

Tissue permeabilization is a pivotal step in the uniform staining of molecules in the whole organ. Nonionic detergents and organic solvents can dissolve lipids, allowing the penetration of antibodies into deep tissue regions [9]. Lipids are not only the basic structural component of neuronal cell membranes but are also present in the axonal *myelin* sheath [10, 11]. In addition, excessive deposition of modified lipids, which are causative factors of aging and neurodegenerative diseases, could restrict antibody accessibility to intracellular antigens [12, 13]. Therefore, suitable permeabilization methods for large-volume imaging are necessary to reconstruct the molecular details of the whole-brain structure at high resolution.

In Alzheimer’s disease (AD), the aggregation of amyloid-beta (Aβ) proteins inside and outside neurons is involved in the dysfunction of synapses, resulting in neuronal malfunctions and the loss of neurons [14, 15]. AD is associated with emotional deficits that manifest as anxiety, apathy, and aggression [16]. Furthermore, hallucinations and psychosis can be observed in approximately one in four to one in three patients with AD [17]**.** Although the majority of synaptic defects in AD have been studied in dendritic spine pathology within interactions of the frontal cortex and hippocampus [18, 19], Aβ pathology also manifests as atrophy of the subcortical neuronal population and neurochemical alterations in the monoaminergic systems [20, 21]. Approximately 35–40% of patients with AD show extrapyramidal symptoms associated with cognitive decline [22, 23]. In addition, the progressive loss of synapses in the nigrostriatal pathway has also been linked to a decline in cognitive and noncognitive symptoms of AD [24, 25]. Dopamine (DA) neurons of AD mice were degenerated at pre-plaque stages, and DA levels were lowered in the hippocampus (HPC) and the nucleus accumbens (NAc), likely contributing to memory loss and mesolimbic cognitive deficits [26–29]. These reports support degenerative changes in DA neurons in AD. However, the structural alterations of the dopaminergic circuits from the brain-wide approach in the AD mouse model are not yet fully understood.

Brain clearing for 3D optical imaging has expanded our knowledge of neural architectures with seamless visualization of neural circuits that cannot be fully described using two-dimensional (2D) approaches [30]. Herein, we developed an enhanced large-scale tissue immunostaining method termed iACT (immuno-active clearing technique). iACT comprises critical steps for epitope recovery and lipid solubilization, namely (i) an aminoalcohol-based surfactant solubilizer, Quadrol, which efficiently removes residual sodium dodecyl sulfate (SDS) in brain tissues after electrophoretic tissue clearing (ETC), which uses SDS for lipid removal; (ii) a zwitterionic detergent, *N*-dodecyl-N,N-dimethyl-3-ammonio-1-propanesulfonate (SB3-12), which enhances antibody diffusion in deep tissue areas without tissue deformation and severe protein loss. This method demonstrated a high-resolution 3D reconstruction across the whole brain. Pathological investigation using iACT revealed alterations in the subcortical structure of 5xFAD mice, including reduced axonal projections of the ventral tegmental area (VTA) neurons into the NAc. Furthermore, we further observed that the axons of VTA-DA neurons failed to reach their targets by swelling and mis-routed targeting the surrounding Aβ plaques, resulting in decreased DA release in the NAc. Overall, iACT provides an optimized protocol for immunolabeling deep neural circuits in the whole brain and reveals impairments of DA pathways in 5xFAD mice.

## Methods

### Mice

All procedures in the mouse experiments were performed in compliance with the protocols approved by the Institutional Animal Care and Use Committee of the Korea Brain Research Institute. The Ai6 (ROSA26-loxP-STOP-loxP-ZsGreen), Ai9 (ROSA26-loxP-STOP-loxP-tdTomato), 5xFAD, Drd1-Cre, Myh11-CreERT2, Thy1-YFP, Nkx2.1-Cre, Tau P301S (PS19), and Rbp4-Cre mutant mouse lines were obtained from Jackson Laboratory. Ai6 and Ai9 Cre reporter mice were used to visualize Drd1-Cre/Nkx2.1-Cre/Myh11-CreERT2, and Rbp4-Cre-expressing neurons, respectively. Tamoxifen (Sigma-Aldrich, *cat.* no. T5648, 4 mg/20 g bodyweight, dissolved in corn oil) was subcutaneously injected a month before sampling to induce Myh11-CreERT2 expression.

### Tissue Fixation

The mice were deeply anesthetized with Avertin and transcardially perfused with 1× PBS, followed by 4% paraformaldehyde (PFA). Brain tissues were dissected, immersed in 4% PFA, and stored at 4 °C overnight. For frozen sections, brain tissues were cryoprotected in 20% sucrose solution overnight at 4 °C and afterward embedded in Optimal Cutting Temperature compound.

### iACT (Brain Clearing and Immunolabeling)

Brain samples were incubated in an acrylamide monomer solution (2% acrylamide/bis solution [29:1, BIO-RAD, *cat.* no. 161-0156]/1% [2,2-Azobis[2-(2-imidazolin-2-yl) propane, Sigma-Aldrich, *cat*. no. 440914] dihydrochloride (Wako Pure Chemical Co., *cat.* no. VA-44) in 0.5x PBS) at 4 °C for 24 h with rocking. Tissues were transferred to sterile 1× PBS in a 6-well plate and polymerized in an X-CLARITY polymerization system (Logos Biosystems, *cat*. no. C30001) at 37 °C and 90 kPa for 3 h. After hydrogel polymerization, the tissues were placed in an ETC chamber (X-CLARITY™ Tissue Clearing System, Logos Biosystems). To extract lipids, 70 V and 1.5 A were applied across the brain tissue with the circulation of ETC Solution (Logos Biosystems, *cat*. no. C130001) through the ECT chamber at 42 °C for 24–48 h. Thereafter, cleared brain tissue samples were washed in PBS three times for 10 min and then incubated in 1% N,N,N′,N′-Tetrakis(2-hydroxypropyl)ethylenediamine (Quadrol) [Sigma-Aldrich, *cat.* no. 122262] at room temperature (RT) with shaking overnight to completely remove SDS.

For immunolabeling, cleared tissues were permeabilized in 4% SB3-12 (Sigma-Aldrich, *cat.* no. 40232) solution at RT for 24 h. Afterward, the samples were incubated at 37 °C for 1–2 days with primary antibodies (Supplementary Table 4) diluted in phosphate-buffered saline with 0.1% Tween™ 20 (PBST). After washing with PBST, tissues were incubated at 37 °C for 1–2 days with secondary antibodies conjugated with Alexa Fluor 488, 568, and 647 (Invitrogen, *cat*. no. A11029, A11003, A21236, A11034 A11037, A31571, A11006 and A11007, 1:500). For routine 3D immunostaining, DeepLabel staining solution (Logos Biosystems; commercialized version of the protocol), based on the iACT method, was used instead of each reagent. To match refractive index (RI) before imaging, cleared tissues were immersed in RI matching solution (50% sucrose [Sigma-Aldrich, *cat*. no. S0389]/25% urea [Sigma﻿-Aldrich, *cat*. no.U5128] in 0.5x PBS) at 4 °C for 24 h.

### Adeno-Associated Virus (AAV) Production

AAV production was performed as previously described [31] although with some modifications. HEK293T cells were maintained in DMEM/F-12 (Gibco, *cat.* no. 11320033)/10% fetal bovine serum (Gibco, cat. no. 26140079) without antibiotics in 150-mm dishes and passaged every 2–3 days. Cells were seeded at 1.5 × 10^7^ 18–24 h before polyethylenimine (PEI) transfection. Afterward, 2–3 mg of the total DNA of RepCap/pHelper/Transfer plasmid (1:1:1 molar ratio):PEI ratio of 1:3 was transfected per plate. Six hours after transfection, the media was replaced. Three days after transfection, cells were scraped, pelleted by centrifugation for 10 min at 2000 g, resuspended in 500 μL of 0.001% Pluronic F-68 (Thermo Fisher Scientific Inc., *cat*. no. 24040-032)/200 nM NaCl (Sigma﻿-Aldrich, *cat*. no. S5886) in PBS per plate, and sonicated four times at 1 s-pulses and 50 amplitudes (Qsonica LLC., *cat*. no. 33-789) for 15 min on ice to lyse the cells. Cell debris was pelleted and collected by centrifugation at 3,220 g for 15 min at 4 °C.

The media were combined with a solution of 40% polyethylene glycol (PEG) 8000 (Sigma﻿-Aldrich, *cat*. no.89510), incubated at 4 °C overnight to facilitate PEG precipitation, and centrifuged at 2818 g for 15 min. The supernatant was discarded, and the pellet was resuspended in 500 μL of 0.001% Pluronic F-68 (Thermo Fisher Scientific Inc., *cat*. no. 24040-032)/200 nM NaCl (Sigma﻿-Aldrich, *cat*. no. S5886) in PBS per plate and mixed with pelleted cell debris. Next, the mixture was incubated at 37 °C, continued for 45 min with 50 units/ml Benzonase® nuclease (Sigma﻿-Aldrich*, cat*. no. E1014)/ 2 mM MgCl2 (Sigma﻿-Aldrich*, cat*. no. M2670), and cell lysates were gently clarified by centrifugation at 2415 g for 10 min at 4 °C. A discontinuous iodixanol gradient was formed by sequentially floating layers: 8 ml of 15% iodixanol in 1 M NaCl and 1× PBS-MK (1 mM MgCl_2_ and 2.5 mM KCl [Sigma﻿-Aldrich*, cat*. no. P5405] in PBS), 6 ml 25% iodixanol in 1× PBS-MK, and 5 ml each of 40 and 60% iodixanol in 1× PBS-MK. Phenol red (0.5%) at a final concentration of 0.125 × 10^−3^% was added to the 15%, 25%, and 60% layers to facilitate identification. Ultracentrifugation was performed using an SW32 Ti rotor in an Optima XE-100 ultracentrifuge (Beckman Coulter Inc., *cat*. no. A94516) at 175,000 g for 2.5 h at 4 °C. Following ultracentrifugation, approximately 4 ml solution was withdrawn from the 40 to 60% iodixanol interface via an 18-gauge needle, dialyzed with PBS containing 0.001% F-68, and ultrafiltered via Amicon Ultra-50 centrifugal filter units with an MWCO column of 100 kDa (Merck Millipore, *cat*. no. UFC5100). After filtration, the AAV stock was aliquoted and stored at −80 °C until use.

### Stereotaxic AAV Injection

For stereotactic delivery of AAVs, the mice were anesthetized by intraperitoneal injection of 1.2% Avertin solution (2,2,2-tribromoethyl alcohol in tert-amyl alcohol [Sigma﻿-Aldrich, *cat*. no. T48402]) dissolved in saline at 200–250 mg/kg body weight. The injection glass capillaries delivered viral vectors at a flow rate of 100 nL/min using a Nanoject Ш (Drummond Scientific). Four-month-old mice were injected with viral vectors unilaterally in the medial prefrontal cortex (mPFC) (AAV-CMV-GFP, AP + 1.8 mm, ML ± 0.9 mm, DV −2.4 mm, relative to bregma), ventral dentate gyrus (vDG) (AAV-CMV-GFP, AP + 0.4 mm, ML ± 2.0 mm, DV −4.2 mm, relative to lambda), and VTA (AAV-pUbC-eGFP and AAV-CMV-Cre, AP − 3.0 mm, ML ± 0.7 mm, DV −4.5 mm, relative to bregma).

### Immunohistochemistry (IHC)

Tissue slices were permeabilized in blocking buffer (4% SB3-12 and 1% bovine serum albumin) at RT for 1 h for immunolabeling. The sections were treated overnight at RT with primary antibodies in the blocking buffer. The slices were then washed three to four times with PBST and incubated at RT for 2 h with Alexa Fluor 488 or Alexa Fluor 568-conjugated secondary antibodies. It was counterstained for 5 min at RT with 1 μg/ml DAPI (Invitrogen, D21490). For quantification of the axonal swelling, axonal swelling was defined by a clear circular outline labeled with Thy1-YFP and TH, measuring over 5 μm in diameter, using the NIS-Elements Advanced Research software (Nikon Instruments). The primary antibody information is provided in Supplementary Table 4.

### Image Analysis and Quantification of the blood vessels

The Imaris software (Oxford Instruments United Kingdon) was used to automatically create 3D rendering and calculate the cross-sectional area of blood vessels. 

### Protein Quantification Using Bicinchoninic Acid (BCA) Assay

The total protein concentration in all steps (after lysis, during washings, and purifications) was calculated using a commercial BCA assay kit (Sigma﻿-Aldrich, *cat.* no. B9643) according to the manufacturer’s protocol. Twenty-five microliters of each sample were mixed with 200 μL of BCA working reagent (50 parts of Reagent A (bicinchoninic acid in 0.1 N NaOH) with 1 part of Reagent B (Copper (II) Sulfate Pentahydrate 4% Solution), kept at 37 °C for 15 min, and then left at room temperature for 10 min to let the temperature even out. Absorbance was read at 562 nm using the FlexStation 3 Multi-Mode Microplate Reader (Molecular Devices LLC, USA). The standard curve was prepared using the BSA standard (Sigma﻿-Aldrich, *cat.* no. P0914).

### Analysis of Residual SDS Removal with Quadrol

To compare the amount of trace SDS remaining in tissue cleared by the ETC process with that removed by Quadrol, we analyzed it using the SDS Detection and Estimation Reagent Kit (G-Biosciences, *cat.* no. 786-129) according to the manufacturer’s instructions. Absorbance was measured at 600 nm using a FlexStation 3 Multi-Mode Microplate Reader (Molecular Devices LLC, USA) in a black 96-well plate.

### Neurochemical Analysis

Coronal brain slices (2 mm thick) were cut (rostral or caudal to the interaural line), and then cortex, dHPC, NAc, DS, and VTA tissue regions were collected by punching with 1 mm diameter Biopsy Punch (Kai Medical, *cat.* no. BP10F). Punched mouse brain slices were sonicated in perchloric acid and centrifuged at 16,000×g, 4 °C for 10 min. The supernatant was used for high-performance liquid chromatography (HPLC) coupled with an electrochemical detector, and the results were analyzed using the Chromeleon 7 software (DIONEX Ultimate 3000 system, Thermo Fisher Scientific Inc.). The samples were separated on a BDS Hypersil C18 column (150 × 3 mm, 3 μM particle size) [Thermo Fisher Scientific Inc., *cat*. no. 30105-052130]. Chromatography was performed at a flow rate of 0.5 mL/min for isocratic elution. The mobile phase (TEST mobile phase, PN: 70-3829) was purchased from Thermo Fisher Scientific Inc., and all standards were purchased from Sigma﻿-Aldrich. Separation and detection were performed at 30 °C. Neurotransmitter levels in the samples were calculated using the area under the curve compared with a standard curve. The neurotransmitter levels were determined as ng/mg protein after normalization.

### Proteomic Analysis

To concentrate the proteins solubilized in PBS and Quadrol solutions, chloroform/methanol purification was performed as previously described [32]. Dried peptides from each sample were resuspended in 20 μL of 0.1% formic acid and analyzed using a nanoliquid chromatography (LC) system (Dionex) and a Q-Exactive Plus Orbitrap mass spectrometer (Thermo Fisher Scientific Inc.). A binary solvent system composed of 0.1% formic acid in water and 0.1% formic acid in acetonitrile was used for all the analyses. Peptide fractions were separated on an Ultimate 3000 RSLCnano System with a PepMap 100 C18 LC column (Thermo Fisher Scientific Inc.) serving as a loading column, followed by a PepMap RSLC C18 (Thermo Fisher Scientific Inc.) analytical column with a flow rate of 0.3 μL/min for 135 min. Full-scan mass spectrometry (MS) with data-dependent MS/MS acquisition was performed in the range of 350–2000 m/z.

All raw liquid chromatography-tandem mass spectrometry (LC-MS/MS) data were processed using the Proteome Discoverer 2.4 (Thermo Fisher Scientific Inc.) by searching for MS2 spectra using the SEQUEST search engine against the UniProtKB/Swiss-Prot mouse database (downloaded on June 17, 2022, containing 84,747 reviewed sequences). The precursor mass tolerance threshold was set at 10 ppm, with a fragment tolerance of 0.02 Da. The false discovery rate for proteins and peptide spectral matches was maintained at 1%. The datasets were normalized using the abundance of total peptides to identify the differentially expressed proteins. The other unmentioned parameters were the Proteome Discoverer default settings.

Accession numbers, normalized abundance values, and related values for high-confidence master proteins were determined using the Proteome Discoverer Software (v2.4) (Thermo Fisher Scientific Inc.), and the values were exported to the R environment (v.4.2.1). Proteins exhibiting significant differences in 1% Quadrol and PBS-washed brain samples were visualized on a scatter plot and pie-donut chart with the ggplot2 package (v 3.3.5) and a heatmap using the ComplexHeatmap package from Bioconductor Release (v3.15). Gene ontology analysis was performed using the Proteome Discoverer Software (V2.4), and their terms were summarized using the dplyr package (v1.0.10).

### Matrix-Assisted Laser Desorption Ionization (MALDI) Mass Spectrometry Analysis

PBS, SDS, and SB3-12 supernatants were obtained sequentially using the iACT procedure. Lipids were extracted using the methanol-chloroform method, as described in a previous study [33]. Briefly, each sample containing lipids was vortexed in a 2:1 chloroform: methanol (v/v) mixture. A tenth volume of water was added, and the mixture was centrifuged at 10,000 × g for 10 min. The organic phase was dried and stored at −80 °C. Prior to MALDI-MS analysis, frozen lipid samples were taken out of the −80 °C freezer and immediately re-suspended in CHCl_3_/MeOH (1:1) and MeOH/H_2_O (2:1). One microliter of each lipid sample was applied to ground steel MTP (Bruker Daltonics, Bremen, Germany). More than six technical replicates of each plate well were prepared during transfer to a 384-well MTP. After air-drying the sample spots, each spot was coated with 5 mg/mL α-cyano-hydroxy-cinnamic acid (CHCA)-matrix in CHCl_3_/MeOH (1:1) supplemented with 0.2% trifluoroacetic acid. In addition, lipid standards (Avanti Polar Lipid Inc., USA) were applied to the ground steel MTP. Lipid profiling was performed in positive and negative ion modes on a MALDI-MS (rapifleX Tissuetyper, Bruker Daltonics) operating in reflection mode. The MS data were acquired using flexControl (v4.0, Bruker Daltonics, Germany) with a mass range of m/z 150–1300.

Raw data were loaded into the R environments (v4.2.1) using the MALDIquant package (v1.21). Mass spectra were preprocessed using square root transformation, TopHat baseline subtraction with a 10% minimal baseline width, and Savitzky– Golay spectrum smoothing. Intensity was normalized using the total ion current calibration method. To correct the calibration differences between the samples, we used the MALDIquant AlignSpectra command. Peaks were picked by applying a signal-to-noise ratio of 5 and a half-window size of 20 and binned via the binPeaks command with a tolerance of 0.05. To avoid matrix effects on lipid analyses, they were excluded from the background peaks confirmed only in the matrix within ±0.1 m/z. All data for the final peaks acquired from MALDI-MS are presented as the mean ± standard deviation.

Afterward, we searched for the final precursor peak lists based on the LIPID MAPS® Structure Database (LMSD) [34]. Lipid species were derived from the filtered peak lists using the LIPID MAPS® REST service of the LIPID MAPS lipidomics gateway, and were determined to be within ±0.05 mass accuracy for [M + H]+, [M + Na]+, [M + K]+, [M − Na]−, and [M − Cl]− potential ion types. An m/z feature was considered significant if *p*-value < 0.01 was obtained. Receiver operating characteristics (ROC) were calculated using the plotROC package (v2.3.0) to evaluate and distinguish selected lipid species. All lipid identifications followed the nomenclature of the LIPID MAPS Lipid Classification System [35]. Finally, our lipidomic data were normalized to internal standards to determine the quantity of over 35 lipid species. Data were transferred to arrays with precursor peaks and normalized intensity values (total ion counts), allowing the direct comparison of samples with different lipid amounts.

### Microscopy and Image Processing

The brain tissue immerged in a urea/sucrose solution to match the RI of the tissue to that of the surrounding medium was imaged using a light-sheet microscope (Ultra microscope, LaVision BioTec, Germany). The light-sheet microscope was equipped with a 2× objective (MVPLAPO 2X, NA = 0.5, WD = 20 mm, Olympus), a SuperK EXTREME EXW-12 pulsed white light laser (NKT Photonics Inc.) and an Andor Neo CMOS camera (Andor, Ireland). The excitation filters of 470/40 and 560/25 were used to excite the Ai6(ZsGreen)/EGFP and Ai9 fluorescent proteins, respectively. The emission filters of 525/50 and 620/60 were used to collect the emitted fluorescence from the proteins. The scan step size was set to 3 μm, and both channels were obtained in two separate scans. For image post-processing, all raw imaging data were collected in TIFF format. 3D-rendered images were acquired using the Imaris software (Oxford Instruments, United Kingdom).

2D images of tissues that had been fixed with 4% PFA were obtained using a confocal microscope (Nikon A1/Ni-E). Fluorescently labeled profiles were analyzed through separate channels using excitation peaks of 405, 488, and 594 nanometers. Consecutive stacks of images were acquired in the septum and NAc regions at high magnification (63×; oil immersion) using Capture Z-Series. Each stack was composed of 1024 × 1024-pixel (8-bit) images with a 1-μm *z*-step. The confocal parameters were set to produce a bright fluorescence signal without saturated pixels. The stack of images was processed with the NIS-Element software.

### Statistical Analysis

All statistical analyses were performed using the GraphPad Prism 9 (GraphPad Software, USA) and R software packages. Unless otherwise noted, differences between two groups were analyzed using the Student’s *t*-test, and multiple comparisons among groups were performed using a one-way ANOVA followed by Tukey’s multiple comparison test.

Differentially expressed proteins were identified with a threshold of ±1.0-fold change over the control and an adjusted *p*-value <0.01. The statistical values of high-confidence master proteins were determined using the Proteome Discoverer Software (Thermo Fisher Scientific Inc.).

## Results

### Effect of Zwitterionic Detergent, SB3-12, on Antigen Retrieval and Tissue Permeability

**Fig. 1:**
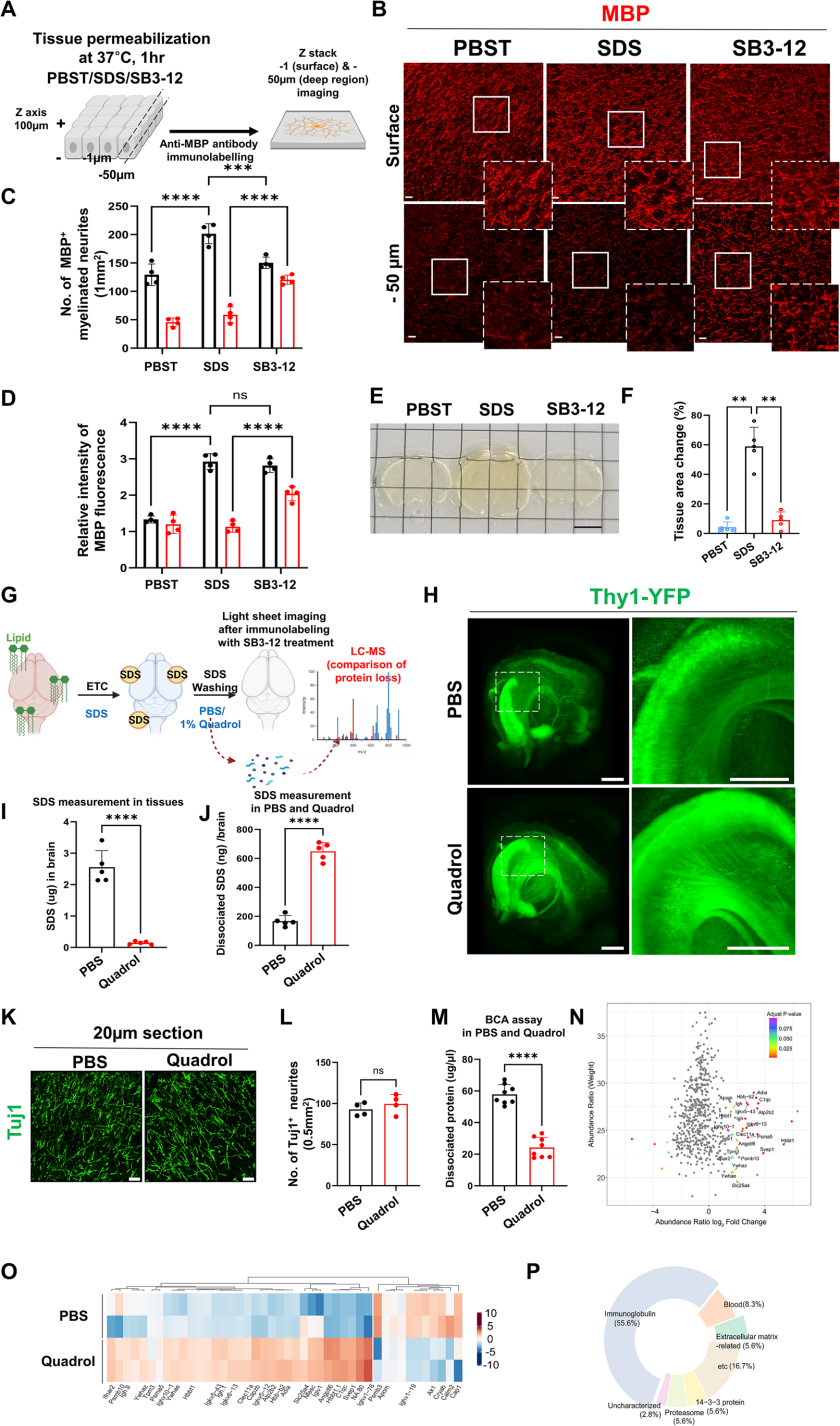
SB3-12 and Quadrol improved brain-wide volume imaging by removing residual detergents and delipidating the brain. **A** Schematic drawing shows a comparison of permeabilizing activities in a 100-μm-thick section by immunofluorescence staining and confocal imaging at 1 and 50 μm *z*-depth for MBP. **B** Representative images of MBP^+^ myelinated neurites in cortical tissues from 4-month-old mice. IHC was performed after incubation of the brain slice with PBST, 4% SDS, and 4% SB3-12 at RT for 24 h. Scale bars: 10 μm. **C** Mbp^+^ myelinated neurites at 1 and 50 μm *z*-depth were quantified. In each condition, images (size 184 μm × 184 μm) were acquired on random fields. **D** Quantification of the Mbp fluorescence signal at 1 and 50 μm *z*-depth. In each condition, images (size 184 μm × 184 μm) were acquired on random fields. **E, F** Representative bright-field images (**E**) and a plot depicting proportions of tissue area changes (**F**) of cleared tissue slices 8 h after PBST, 4% SDS, and 4% SB3-12 treatment. Scale bar: 5 mm. **G** Schematic representation of light sheet imaging and dissociated proteome analysis in ETC brain-washed PBS or 1% Quadrol after ETC. A schematic drawing was created with https://www.biorender.com/. **H** Light sheet microscopic imaging of fluorescence signals from the cleared brains of Thy1-YFP mice washed with PBS or 1% Quadrol for 24 h. Scale bars: 2000 μm. **I** Quantification of residual SDS in tissues after washing with PBS or 1% Quadrol. **J** Quantification of dissociated SDS in PBS or 1% Quadrol. **K, L** Representative confocal images (**K**) and quantification (**L**) of Tuj1^+^ neurites in cortical sections incubated with PBS or 1% Quadrol for 24 h at RT Scale bars: 20 μm. **M** A plot depicts the quantification of dissociated protein concentration from brain tissues washed with PBS or 1% Quadrol. **N** A scatter plot shows the weighted abundance ratio and log_2_ abundance fold change scatter of proteins obtained from 1% Quadrol-washed and PBS-washed brain samples. The *X*-axis represents the average log_2_ (total intensity normalized abundance + 1). The proteins in the colored gradient red or blue had an adjusted *p*-value <0.1 and an abundance fold change > 2. **O** Heatmap of log_2_-centered normalized abundance for 44 differentially expressed proteins. Each row represents a technical replicate (*n* = 2) of 1% Quadrol-washed and PBS-washed brain samples. The gradient color on the right bar indicates a log2-centered normalized abundance value. **P** Donut schematic diagram depicting Gene Ontology analysis of the proteins enriched in 1% Quadrol-washed ETC brain samples. The terms from the GO cellular component revealed that the reliably identified proteins in 1% Quadrol-washed ETC brain samples were involved in blood tissues or vessels

Antibody diffusion into tissue samples is a rate-limiting step for immunostaining thick specimens. To overcome the inefficiency of passive diffusion of antibodies in thick tissue samples, we first tested diverse detergents and found that zwitterionic detergents could increase permeabilization of brain tissues. Zwitterionic detergents efficiently dissolve membrane lipids because of their higher critical micelle concentration (CMC) [36, 37], resulting in improved non-degradative epitope recovery and membrane solubilization in thick tissues. These features lead to better performance of tricky antibodies, such as those that recognize subcellular structures and nuclear proteins [38]. Furthermore, owing to the lack of net charges on the hydrophilic head groups, zwitterionic detergents have a low denaturing property, which might protect the native state of proteins [39, 40]. To investigate the efficacy of zwitterionic detergent SB3-12 in facilitating antigen retrieval and antibody permeabilization, we compared the immunolabeling efficiency using 100-μm-thick mouse brain slices treated with detergents such as 0.1% PBST, 4% SDS, and 4% SB3-12 for tissue permeabilization (Fig. 1A). SDS treatment prior to IHC yielded the highest degree of efficiency in antibody staining of MBP (myelin basic protein) protein on the tissue surface (Fig. 1B-D). However, neither PBST nor SDS penetrate the antibody to a depth of 50 μm. Pre-treatment with SB3-12 resulted in not only robust immunostaining on the surface but also efficient penetration of the antibody deep into the tissue, which allowed high-resolution fluorescence imaging of MBP^+^ and Tuj1^+^ (neuron-specific class III beta-tubulin) cells in deep tissue areas (Fig. 1B–D; Supplementary Fig. 1A-1C). Therefore, while SDS tangles antibodies on the surface, SB3-12 effectively retrieves antigen and permits tissue permeabilization.

To evaluate whether SB3-12 is suitable for immunolabeling cleared brains, we assessed the alteration of tissue transparency and size deformation on 1-mm cleared brain tissue slices by permeabilizing detergents. The degree of tissue deformation was quantified by measuring tissue expansion. SDS caused significant deformations in the cleared tissues, and furthermore, it interfered with the proper RI matching compared with Tris-buffered saline with 0.1% Tween™ 20 (TBST)-treated control tissue (Fig. 1 E and F). Consequently, this interference led to an insufficient attainment of tissue opacity. In contrast, we found that SB3-12 effectively conserved tissue integrity and exhibited no detrimental impact on the tissue clearing process (Fig. 1 E and F). SB3-12 has a shorter hydrophobic tail and consequently forms a higher CMC (3 mM) [41]. SB3-12 refolds a denatured form of Tom40 containing inclusion bodies [42] and successfully solubilizes membrane proteins [43, 44]. These findings, together with our results, indicate that SB3-12 may efficiently disrupt hydrophobic-hydrophilic interactions of cellular components and has the potential to achieve effective antibody staining for volumetric imaging without affecting the tissue clearing process.

### Effect of Quadrol on Residual SDS from ETC-Cleared Brain Without Severe Protein Loss

ETC is the most robust and rapid tissue-clearing method; however, it uses SDS to extract lipids from tissues. Although the tissue was washed in PBS for 1–2 days to remove SDS, residual SDS remained in the cleared tissue, which caused light scattering and absorption. The residual SDS may also inhibit the antibody-antigen binding reaction, thereby impeding the immunostaining of the ETC-cleared brain [45]. We noted the tissue-solubilizing activity of the aminoalcohol, Quadrol. The cationic amino groups of Quadrol could contribute to the solvation of anionic surfactants. We observed that ETC-cleared brain tissues in a cold chamber showed white precipitation of SDS, whereas Quadrol treatment of ETC-cleared tissues showed transparent brain tissues without SDS precipitation (data not shown). Thy1-YFP mice carrying the Thy1 promoter-driven cytosolic fluorescent YFP have been used for volumetric brain imaging with strong green fluorescence signals [46, 47]. To test whether Quadrol can be applied to ETC-cleared tissues to obtain better volume imaging, we performed light-sheet imaging of 1% Quadrol-washed ETC-brain hemispheres from Thy1-YFP mice (Fig. 1G). Quadrol washing after ETC facilitated deep tissue immunolabeling, resulting in a clear visualization of hippocampal axon bundles (Fig. 1 H). After Quadrol washing, we measured SDS amounts of brain tissues and Quadrol. Quadrol washing led to a substantial reduction of SDS content within cleared brain samples (Fig. 1I). Consistent with this, residual SDS was prominently present in the Quadrol solution (Fig. 1J). The treatment of Quadrol prior to immunolabeling coronal brain sections demonstrated that Quadrol did not have an impact on the antibody reaction (Fig. 1 K and L). To investigate whether the immersion of ETC-cleared tissues in Quadrol solution could affect tissue integrity alongside severe protein loss, we performed a BCA assay and proteomic analysis with liquid chromatography-mass spectrometry (LC-MS) using dissociated proteins in Quadrol and PBS solutions (Fig. 1G). The number of proteins dissociated from cleared tissues was dramatically reduced by Quadrol washing, indicating the successful removal of SDS (Fig. 1M). Proteomic analysis revealed the Quadrol solution contained 44 additional solubilized proteins compared to the PBS solution (Fig. 1N, O; Supplementary Table 1). Approximately 64% of these proteins consisted of immunoglobulins and blood cell proteins, which were supposed to be remnants of circulating blood cells and remained in the brain tissue after perfusion (Fig. 1P). Thus, Quadrol may be adapted to ETC-mediated brain clearing methods to efficiently preserve fluorescence signals by removing residual SDS signals without severe protein loss.

### Optical Characterization of Fine Neural Processes via the Application of iACT in Model Mice

**Fig. 2:**
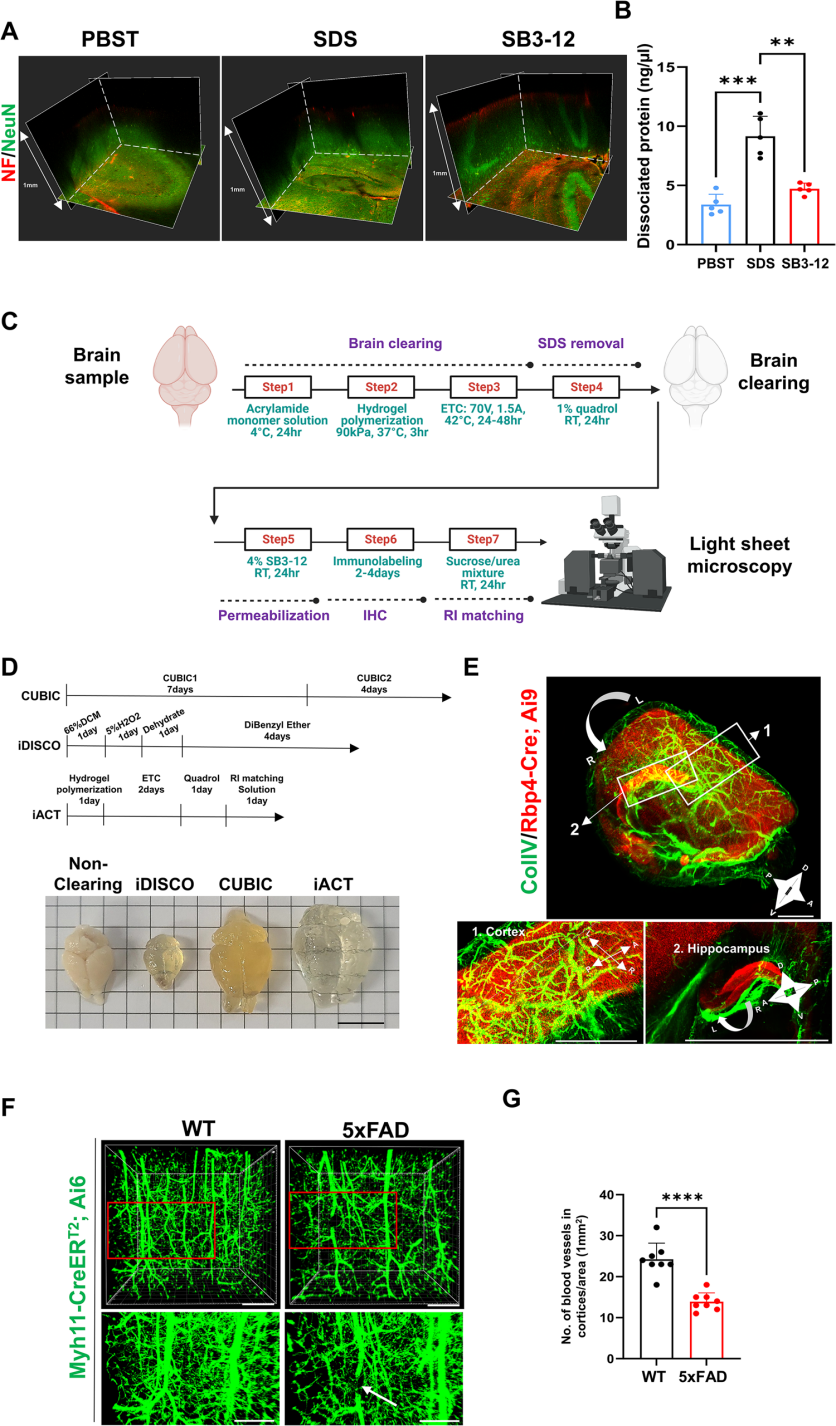
Immuno-active clearing technology (iACT) is efficient and compatible for neuropathological assessment of the mouse brain. **A** *X*, *Y*, and *Z* axis-plane images of the mouse brain fluorescently labeled with NF and NeuN after pretreatment with PBST, SDS, and SB3-12. **B** BCA assay of dissociated proteins from ETC brains after washing with each detergent for 24 h RT. **C** Schematic diagram of the immuno-active clearing technique (iACT) protocol. (1) Brain tissue clearing by ACT. (2) Residual SDS removal by washing with 1% Quadrol. (3) Incubation in 4% SB3-12 for tissue permeabilization, followed by immunolabeling. (4) Immersion in sucrose/urea solution for RI matching. (5) Light sheet microscopic imaging. **D** (upper schematic) Timeline of the CUBIC, iDISCO, and iACT methods for the tissue clearing process. (Bottom image) Representative bright-field images obtained by the CUBIC, iDISCO, and iACT methods to compare the transparency of aged 5xFAD mouse brain tissue. Scale bars: 1 cm. **E** iACT-facilitated volume imaging of Rbp4-Cre-induced tdTomato Cre reporter fluorescence in the Rbp4-Cre; Ai9 (ROSA26-loxP-STOP-loxP-tdTomato) mouse brain hemisphere immunostained for anti-ColIV antibodies. Scale bars: 2000 μm. Representative light sheet microscopic images (**F**) and quantification (**G**) of blood vessels in the cortical regions of iACT-performed brains from Myh11-CreER^T2^;Ai6;WT and Myh11-CreER^T2^;Ai6;5xFAD mice. Scale bars: 300 μm

After delipidation by ETC, we compared detergents to find the most efficient tissue-permeabilizing solution. TBST was insufficient to allow antibodies to penetrate deep into tissue (Fig. 2A). While an SDS antigen retrieval solution can be used on PFA-fixed tissues, prolonged and continuous use of SDS caused severe protein loss (Fig. 2B) making it difficult to reflect the correct structure (Fig. 2A). SB3-12 treatment did not cause protein loss and achieved intact immunostaining deep into the brain tissue (Fig. 2 A and B). SB3-12 permeabilization could be applied even to 10-μm-thin sections to enhance epitope retrieval without tissue deformation (data not shown). These results suggest that SB3-12 treatment could be used as a universal antigen retrieval step, including thick brain tissues. Collectively, we optimized the previously reported ACT protocol, which was developed to enhance ETC-based clearing methods [48]. We added a Quadrol washing step after ETC, followed by an SB3-12 permeabilization step to remove residual SDS and increase tissue permeability. We used a urea/sucrose mixture, reagent 2 of the CUBIC protocol [47] for RI matching. The entire iACT procedure, shown in Fig. 2C, facilitated efficient antibody labeling for whole-brain optical imaging within a week.

Fluorescent reporter transgenic mice have been widely used for understanding brain development and the etiology of neural diseases, and 3D imaging of fluorescent reporter mice could provide unforeseen phenotypes. We examined the application of iACT to diverse transgenic reporter mice in combination with antibody labeling. First, iACT was performed on the brains of 4-month-old Thy1-YFP mice. Z-stack images of the sagittal sections of the brain hemispheres were collected in the medial to lateral direction. Intact neuronal structures were visualized in different regions, including the frontal cortex, hippocampus, and striatum (Supplementary Fig. 2A). To apply iACT to the brains of Cre-reporter mice, we crossed Nkx2.1-Cre mice with Ai6 Cre reporter mice (Supplementary Fig. 2B). Nkx2.1 is required for the specification of GABAergic interneurons in the subcortical telencephalon [49]. In the adult mouse brain, Nkx2.1-Cre is strongly expressed in the bed nucleus of the stria terminalis (BNST) and hypothalamus (HY). These Cre-dependent ZsGreen-positive neurons were clearly distinguishable from anti-tyrosine hydroxylase (TH) antibody-labeled DA neurons (Supplementary Fig. 2B). Therefore, iACT accelerated a variety of genetic approaches for subcellular-level optical analysis and unbiased exploration of whole neural structures of interest with antibody labeling.

Genetically encoded fluorescent proteins become less fluorescent after tissue fixation [47]. Therefore, efficient immunolabeling for volumetric histological imaging is required to obtain sufficient signals from cleared tissues that harbor fluorescent reporters. AAV-mediated neural circuit tracing requires seamless rendering of neural circuits, which is impossible to achieve using serial imaging of thin sections. However, AAV tracers are not as bright as transgenic reporters such as Thy1-YFP, and 3D imaging of AAV tracers is hardly accessible. We evaluated the performance of SB3-12 incubation in detecting fluorescence signals of AAV2.retro-CMV-EGFP [50] by injecting AAV into the vDG of 4-month-old C57BL/6 mice. Application of iACT clearly visualized retrograde labeling of viral EGFP from the vDG to the entire hippocampal circuitry (entorhinal cortex-hippocampus [vDG-CA3-CA1-subiculum-septum-mammillary body]), whereas EGFP signals were mostly detected in the AAV injection site (vDG) with non-labelling control (Supplementary Fig. 2C). We successfully visualized large axon bundles in the fornix (Supplementary Fig. 2C). These results indicate that immunolabeling is a prerequisite for the effective amplification of signals from fluorescent reporters in an aging brain and greatly improves neural circuit mapping using viral tracers. Next, iACT was performed for anterograde tracing experiments to visualize the thin axonal projections. Intracerebral AAV-carrying fluorescent gene injection to specific areas provides valuable information about the neural circuitry [51]; however, it was hampered by weak axonal signals that render thin individual axonal projections. We unilaterally delivered AAV-CMV-GFP [52] into the mPFC of 4-month-old C57BL/6 mice. Two weeks later, iACT with anti-GFP antibody staining permitted the acquisition of clear images of axonal innervation through the NAc, basolateral amygdala (BLA), and VTA (Supplementary Fig. 2D). Each individual axon was brightly labeled and successfully provided information on seamless axonal projections. Collectively, iACT successfully achieved 3D volumetric imaging of cleared adult brains harboring endogenous fluorescence reporters and neural circuits labeled with viral traces.

### Application of iACT for the Neuropathological Investigation of in an AD Mouse Model

Most methodological approaches to 3D optical clearing have been performed using young adult mouse brains. However, it has remained challenging to achieve homogeneous imaging of the aged whole brain because of large lipids and molecular barriers. The aged 5xFAD mice, which recapitulate five familial AD (FAD) mutations [53] exhibit abnormal lipid metabolism, resulting in excessive lipid deposition within the brain. We evaluated the aged tissue clearing capability of iACT. Comparison of iACT and other whole brain clearing methods, including iDISCO and CUBIC, revealed that iACT most efficiently and rapidly achieved complete brain transparency in 6-month-5xFAD mice (Fig. 2D). Given the successful visualization of the AD mouse model, we performed iACT to investigate both cellular and structural attributes associated with AD. Six-month-old PS19 tauopathy mice carrying the Thy1-YFP reporter have been used to demonstrate tauopathy-related axonal spheroids [54]. We applied ACT-based clearing to PS19; Thy1-YFP mouse brains to visualize YFP-labeled neurons. Visual inspection of ACT imaging revealed low endogenous fluorescence signals, displaying remarkable neuronal cell bodies and indistinguishable dendrites autofluorescence from blood vessels also affects volumetric brain imaging (Supplementary Fig. 3A, upper panel). The application of iACT to the PS19; Thy1-YFP mouse brain using anti-GFP antibody labeling resulted in significant improvement of Thy-YFP signals in neuronal processes and displayed phagocytic engulfment of neuronal Thy-YFP by glial cells (Supplementary Fig. 3A, bottom panel) providing evidence that microglia excessively prune tau positive synapses [55].

Brain vasculature networks are indispensable components of neural processing units known as neurovascular units and are solely responsible for the transportation of vital nutrients and blood cells [56]. A detailed characterization of 3D brain vasculature will give a chance for a brain-wide investigation of the blood-brain barrier dysfunction in neurological disorders. iACT was applied to the Rbp4-Cre; Ai9 mouse brains using an anti-ColIV antibody and provided precise 3D imaging of physical relationships between tdTomato (Ai9)-positive pyramidal neurons and cortical and hippocampal vascular structures (Fig. 2E). These findings led us to investigate vascular damage in an AD mouse model using iACT. Myh11-CreER^T2^;Ai6 fluorescent reporter mice showed Cre-dependent, tamoxifen-inducible labeling of blood vessels in the brain with ZsGreen. To examine whether SB3-12 facilitates the elucidation of the pathogenesis of 5xFAD mice, we applied iACT protocols to the Myh11-CreERT2;Ai6;WT and Myh11-CreERT2;Ai6;5xFAD mouse brains. We found that there was capillary leakage (Fig. 2F, white arrow) and a significant decrease in the number of Myh11-CreERT2-labeled brain blood vessels (Fig. 2 F and G). Taken together, our SB3-12-based iACT protocol is suitable for the pathological investigation of neurodegenerative disease models.

### SB3-12 Exhibits a Specific Role in the Process of Lipid Removal

**Fig. 3:**
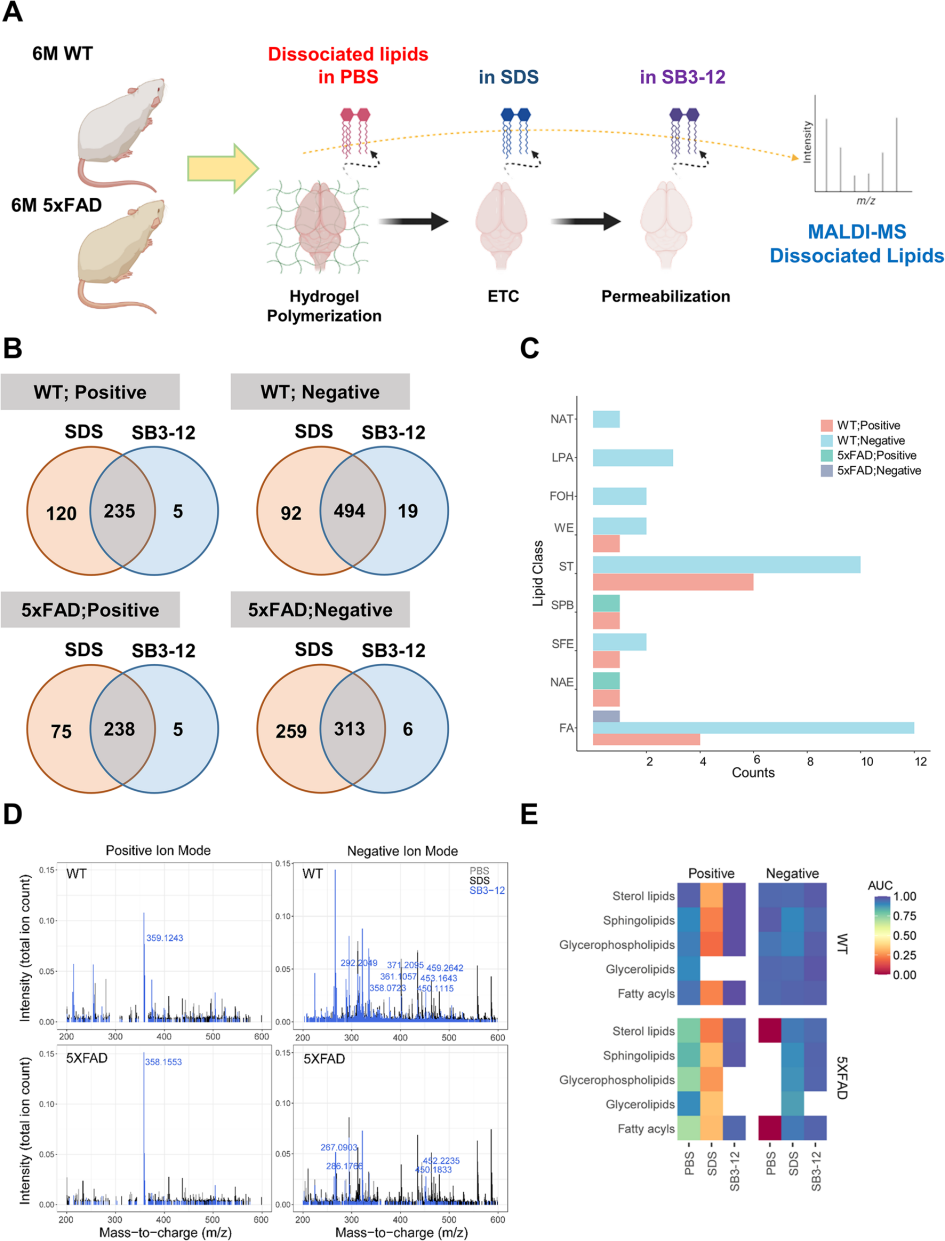
SB3-12 effectively removes lipid species in the mouse brain in 3D volume imaging of the mouse brain. **A** Schematic diagram of lipidomic analysis in PBS, SDS, and SB3-12 supernatants obtained during iACT processing of brains from 6-month-old WT or 5xFAD mice. **B** Venn diagrams depict the overlaps between the peak types identified in the mass spectra when MALDI-MS was applied to solutions obtained by treating WT and 5xFAD polymerized brains with SB3-12 and SDS in both cationic and anionic modes. **C** Using the LIPID MAPS database, a lipid search was conducted for each peak that exhibited a significant increase in total ion count. The search results were visualized with the appropriate lipid subclasses in a bar graph. **D** Identified peaks of the most abundant expected lipid species within averaged mass spectra between m/z 300–1000 obtained from MALDI-MS at positive and negative ion modes. Each peak displayed the distribution of lipids between WT and 5xFAD mice across three washing steps and positive/negative ion modes. Bin width = 0.1 m/z. **E** A heatmap-visualized receiver operating characteristics (ROC) analysis of five lipid species results in the identification of solubilized lipids by each wash step. Area under the ROC curve (AUC) values were obtained from lipids significantly detected in each ion mode (*p* < 0.01). The color scale in the heatmap indicates the AUC values of each lipid species

We investigated whether SB3-12 facilitates lipid extraction to enhance the access of antibodies to intracellular antigens. Acrylamide-polymerized 6-month-old WT or 5xFAD mouse brain tissues were subjected to stepwise treatment with PBS, SDS, and SB3-12. Supernatants were collected after each treatment and analyzed using MALDI-MS (Fig. 3A). The workflow consisted of spotting supernatants on the MALDI target plate with the CHCA matrix, air-drying the solvents, and analyzing them with MALDI-MS. MALDI-MS analysis of SDS-treated and SB3-12-treated WT mouse brains revealed that 355 and 240 peaks were detected in the positive mode, respectively (Fig. 3B). In the negative mode, 586 and 513 peaks were detected in SDS-treated and SB3-12-treated WT mouse brains, respectively. Similarly, the MALDI-MS analysis of 5xFAD mouse brains treated with SDS and SB3-12 revealed that 313 and 243 peaks were detected in the positive mode, respectively. In the negative mode, 572 peaks were detected in SDS-treated 5xFAD mouse brains, while 319 peaks were detected in SB3-12-treated 5xFAD mouse brains.

Further analysis revealed that 235 and 238 peaks overlapped between the two samples in the positive mode in WT and 5xFAD mice, respectively. In the negative mode, 494 and 313 peaks overlapped between the two samples in WT and 5xFAD mice, respectively. To investigate which lipid classes were removed by SB3-12 treatment in the brains of WT and 5xFAD mice, we calculated the fold change and *p*-value of the total ion count values of the SB3-12-treated samples compared to the total ion count values of the SDS-treated samples for each of the overlapping peaks. A log_2_ fold change value greater than or equal to 1 indicates that the peak was more abundant in the SB3-12-treated sample than in the SDS-treated sample. A *p*-value of less than or equal to 0.00001 indicates that the difference in abundance between the two samples is statistically significant. In the mass spectrum of the WT mouse brain sample, 24 peaks in the positive ion mode and 43 peaks in the negative ion mode met these criteria. This means that the abundance of these peaks was significantly increased in the SB3-12-treated sample compared to the SDS-treated sample. In the mass spectrum of the 5xFAD mouse brain sample, two peaks were found in the positive ion mode and 11 peaks were found in the negative ion mode. The filtered peak lists were then submitted to the LIPID MAPS database to search for appropriate lipid subclasses. The search was conducted within a 50-ppm mass tolerance of each m/z value for the potential ion types [M + H]+, [M + Na]+, [M + K]+, [M − H]−, and [M − Cl]−. The search revealed that the following lipid classes were more removed from polymerized brain by SB3-12 than by SDS: NAT (N-acyl taurines), LPA (lysophosphatidic acid), FOH (fatty alcohols), WE (wax esters), ST (sterols), SPB (sphingoid bases), SFE (short fatty esters), NAE (N-acyl ethanolamines), and FA (fatty acids) (Fig. 3C).

Of the peaks from the WT mouse brain, a positive peak of m/z 359.1243 and seven negative peaks were detected only in the samples treated with SB3-12, in contrast to those of PBS or SDS (Fig. 3D). Next, we examined how efficiently SB3-12 removed lipid species from the 5xFAD mouse brain. We analyzed the mass spectra of SB3-12 supernatants after treatment of cortical tissues from WT and 5xFAD mice. There was a unique peak of m/z 358.1533 in the positive modes and four peaks of m/z 267.0903, 286.1766, 450.1833, and 452.2235 in the negative ion modes, distinguished from those of SB3-12 supernatants obtained from the WT mouse brain (Fig. 3D). Treatment of the 5xFAD mouse brain with SB3-12 led to the detection of a negative peak of m/z 450.1813, which is different from the SDS supernatant. The negative peak of m/z 267.0903 was exclusively found in the SB3-12 supernatants of the 5xFAD mouse brain. The precursor peaks were searched in the LIPID MAPS structure database within ±0.05 Da tolerance [34]. The putatively identified lipid candidates exclusively acquired by SB3-12 and from 5xFAD brain tissues with SB3-12 solubilization are summarized in Supplementary Tables 2 and 3.

Based on the analysis of the mouse brain lipidome specifically solubilized by SB3-12, a question of interest arose to determine how well the analyses were discriminative between or within samples at specific levels of normalized intensity (total ion counts). The area under the curve (AUC) values were calculated using samples, ion adducts, and washing steps. The estimated AUC values for the lipid species suggested that the analysis of lipids extracted by SB3-12 treatment was close to perfect discrimination (Fig. 3E). Except for glycerolipids, the AUC values for lipids extracted from WT and 5XFAD mouse brains showed an obvious downward trend after SDS treatment in the positive ion mode. SB3-12 supernatants from WT and 5xFAD mouse brains showed an increasing trend in AUC values for fatty acyls, sphingolipids, and sterol lipids. The values in the negative ion modes showed similar tendencies in the overall lipid species and WT/5xFAD mouse brains. The three kinds of lipid species from WT mouse brains solubilized by SB3-12 treatment, matched with a precursor peak of m/z 359.1243 at positive ion modes, had high AUC values, such that they were distinguished from those by an SDS washing step. AUC values for the WT mouse brain in the negative ion modes revealed that all lipids matched with seven precursor peaks were also discriminative within each lipid species. In addition, the lipid species matched with a positive peak and four negative peaks extracted from the 5xFAD mouse brain solubilized by SB3-12 were more discriminative than SDS or PBS supernatants. These results suggest that SB3-12 is specifically involved in the removal of lipids, which is a putative mechanism for antibody penetration in adult mouse brain tissues or AD model mouse brains.

### iACT Precisely Provides 3D Optical Information of Midbrain Neural Circuits in an AD Mouse Brain

**Fig. 4:**
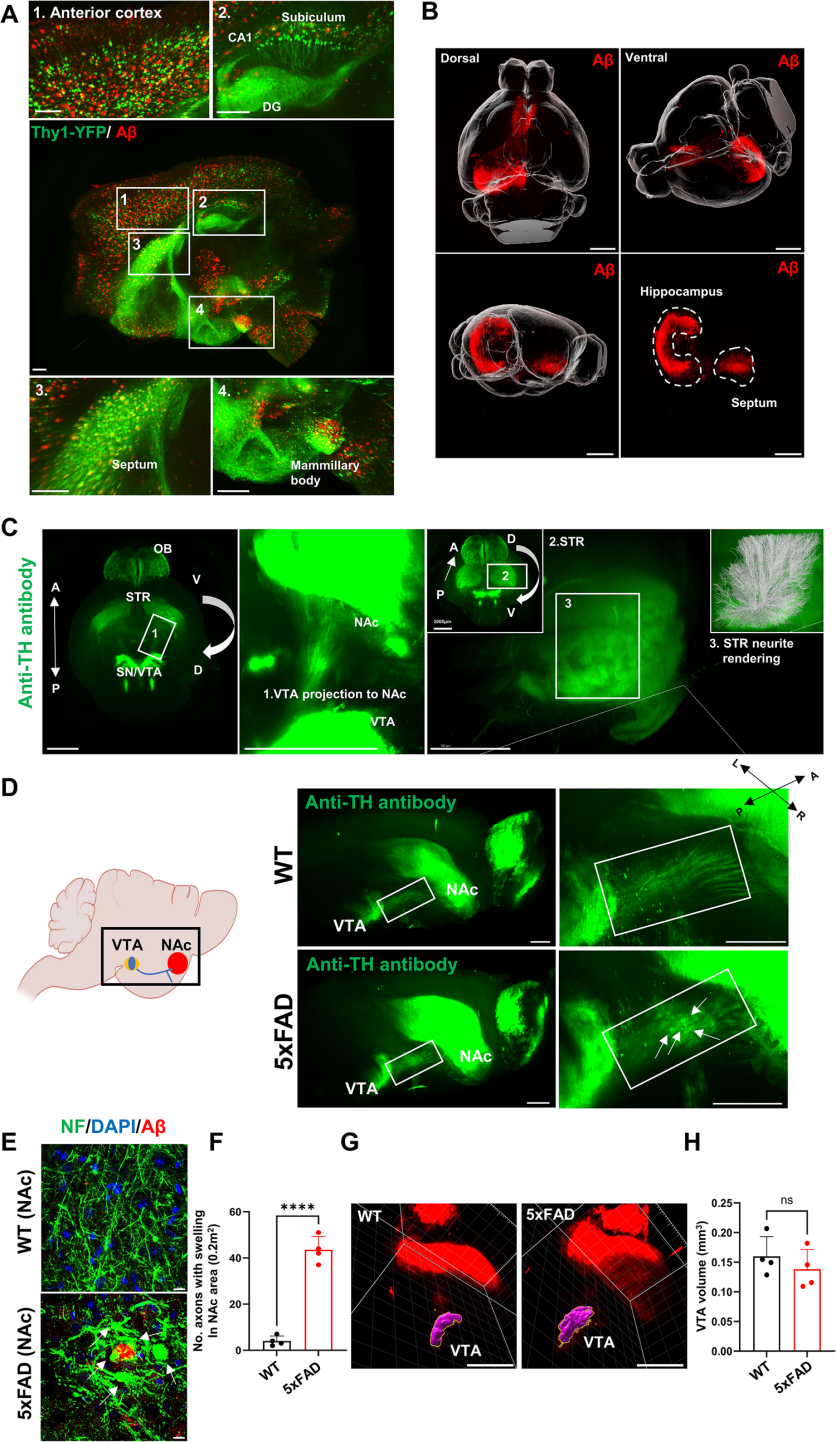
iACT-assisted visualization of DA neural circuits and their structural abnormalities. **A** Optical 3D imaging acquired on a light-sheet microscope from a 6-month-old 5xFAD; Thy-YFP mouse brain labeled with GFP and Aβ plaques. DG: dorsal dentate gyrus. Scale bars: 700 μm. **B** Reconstruction of the mouse brain with a 3D rendering of amyloid deposition in the septo-hippocampal pathway. Scale bars: 3000 μm. **C** Visualization of whole mouse brain stained with an anti-tyrosine hydroxylase (TH) antibody by iACT. OB, olfactory bulb; STR, striatum; SN/VTA, substantia nigra/ventral tegmental area. Scale bars: 2000 μm. **D** Schematic diagrams of iACT analysis of the mesolimbic pathway (left) and 3D clearing tissue image (right) of the iACT-cleared brain tissue in 6-month-old female WT or 5xFAD mice as representatives of TH expression. VTA, ventral tegmental area; NAc, nucleus accumbens. Scale bars: 1000 μm. **E** Representative immunofluorescence microscopy images of neurofilament (NF) and Aβ in NAc of 6-month-old WT/5xFAD mice. Scale bars: 10 μm. **F** Quantification of axons with swelling phenotypes in NAc of 6-month-old WT/5xFAD mice. NF staining was used to quantify axonal swelling in the NAc of 6-month-old WT or 5xFAD mice. Images (size 130 μm × 130 μm) were acquired on random fields, and axonal swelling (above 5 μm in diameter) was selected from areas around Aβ plaques. **G** Representative 3D images of the region of interest (ROI, pink) for the VTA tissues from 6-month-old WT and 5xFAD mice. Scale bars: 2000 μm. **H** The VTA volume of wild-type and 5xFAD 6-month-old mice was quantified using Imaris

We evaluated whether iACT enabled accurate mapping of amyloidosis. iACT revealed that Aβ aggregates were observed throughout the subregions, including the cortex, hippocampus, and hippocampal memory circuits (mammillary body, septum, and subiculum) in the brain of 4-month-old 5xFAD mice (Fig. 4A). The septo–hippocampal pathway is connected to CA1 network excitability, which is important for representing distinct behavioral states via theta rhythmogenesis [57]. AD model mice have shown prominent alterations in the septo-hippocampal pathway, with a reduction in cholinergic cell size [58]. iACT clearly showed that Aβ plaques were widely deposited in the septo-hippocampal pathway, providing optical evidence linking AD pathology with hippocampal-septal circuits of 4-month-old 5xFAD mice (Fig. 4B).

Next, we generated a complete 3D map of TH^+^ neurons using a 4-month-old WT (C57BL/6) mouse whole brain. iACT permitted strong TH labeling of DA pathways, including the VTA and terminal projection areas in the NAc (Fig. 4C). In particular, iACT provides a patched surface shape made from DA axonal endings in the striatum (STR) (Fig. 4C-3). Alterations in the DA system are frequently reported in patients with AD, contributing to age-related memory decline and non-cognitive symptoms [28, 59]. VTA DA neurons project to various limbic and cortical brain regions [60]. 3xTg-AD mice showed an augmentation in the density of postsynaptic D2/3R receptors within the STR, along with an increase in D2/3 autoreceptors present in the cell bodies of the substantia nigra (SN) and VTA [61]. An electroencephalogram (EEG) study using a 5xFAD mouse model has indicated that disrupted DA neurotransmission contributes to enhancements in delta and alpha oscillations, while beta activity is attenuated [62]. Furthermore, DA plays a crucial role in maintaining normal motor control. In the context of 5xFAD mice, motor dysfunction is evident, as confirmed by deficits observed in behavioral tests [63, 64]. Motor deficits in 5xFAD mice become evident between 6 and 9 months of age. To assess potential impairments in DA neural circuits, we performed iACT for whole-brain immunolabeling on 6-month-old 5xFAD mice and age-matched WT mice with anti-TH antibodies. We observed swollen axons in the VTA neurons in the 5xFAD mice (Fig. 4D). Immunostaining on coronal sections offers precise visualization of axonal swellings surrounding Aβ plaques within NAc (Fig. 4 E and F). These discernible swellings serve as indicators of aggregated axonal terminals originating from VTA DA neurons in 5xFAD mice. The utilization of 3D volumetric imaging by iACT provides a means to quantify tissue volume, a task that becomes arduous when employing traditional 2D images. Although there was aggregation of DA neuron axons in the NAc region, the volume of the VTA tissue remained unchanged (Fig. 4 G and H).

### Amyloid Pathology is Closely Associated with Disruption of DA Pathway

**Fig. 5:**
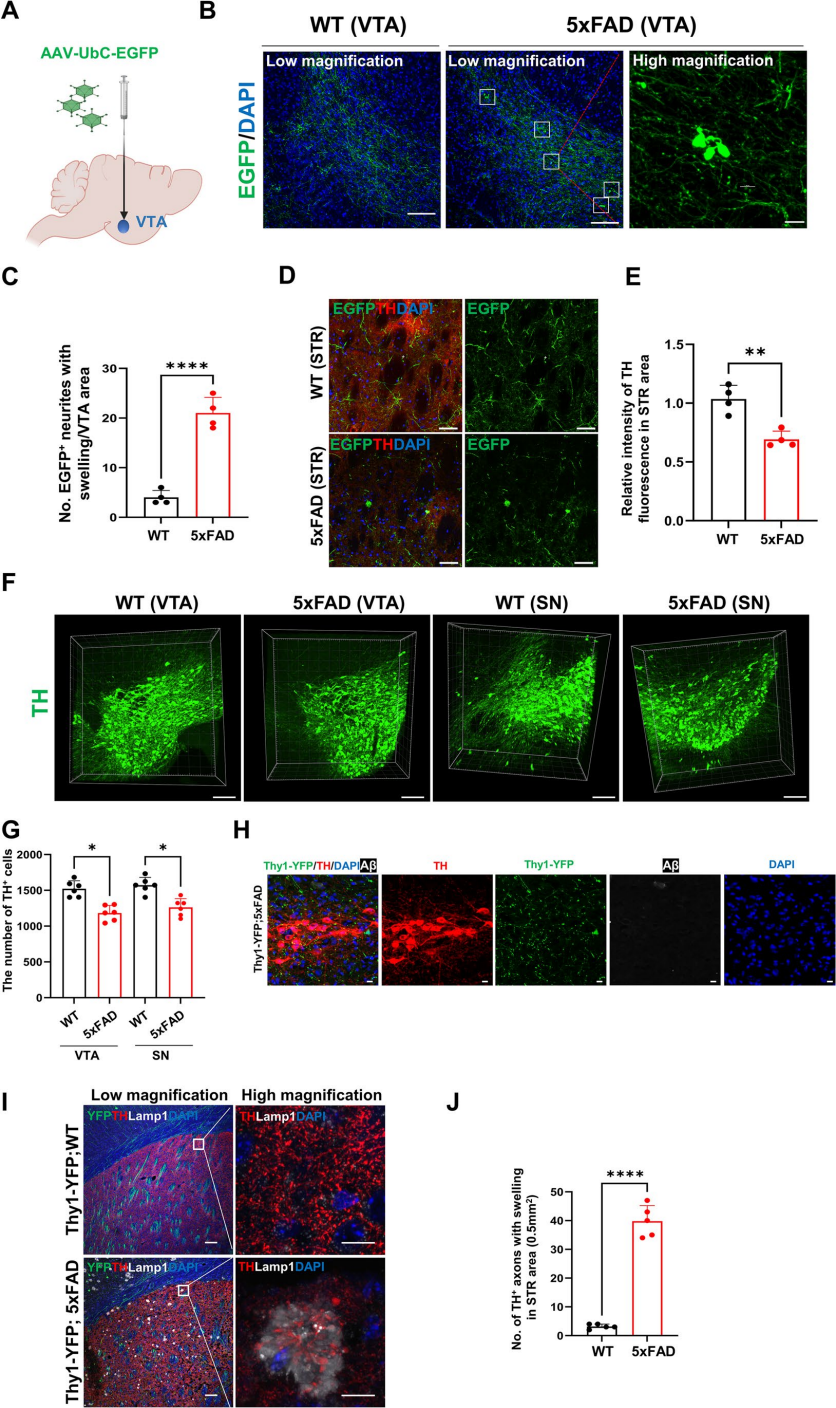
Axonal swelling of DA neuronal axons within the VTA and septum. **A** Schematic diagram of stereotaxic injection with AAV carrying pUbC-EGFP to VTA. **B** Representative EGFP immunofluorescent images of the VTA area from 4-month-old WT or 5xFAD mice that were injected with AAV carrying pUbC-EGFP into the VTA. Scale bars: 100 μm (low magnification) and 10 μm (high magnification). **C** Quantification of EGFP^+^ VTA axons with swelling phenotypes in 4-month-old WT/5xFAD mice injected with AAV carrying pUbC-GFP into the VTA. **D** Representative confocal images for EGFP and TH in the STR area of WT/5xFAD mice. Scale bars: 50 μm. **E** Measurement of the TH signal indicates abrogation of DA release from VTA to STR in 6-month-old 5xFAD mice. **F** 3D visualization of TH-labelled DA neurons in VTA and SN of the midbrain from 6-month-old WT and 5xFAD mice using the iACT method. Scale bars: 200 μm. **G** Quantification of TH^+^ cells through 3D rendering of TH-labeled somas in VTA and SN regions of 1-mm-thick midbrain tissues using the Imaris software. **H** The visualization of TH in the VTA of Thy1-YFP;5xFAD mice at 6 months revealed that YFP fluorescence was not detected in TH^+^ DA neurons. Scale bars: 10 μm. **I** Representative images for Thy1-YFP/TH (left panel) and Lamp1/TH (right panel) in the STR area of 4-month-old Thy1-YFP;WT and Thy1-YFP;5xFAD mice. Scale bars: 100 μm (low magnification) and 10 μm (high magnification). **J** Quantification of TH^+^ axonal terminal aggregations in the STR areas of WT and 5xFAD mice

We investigated early structural changes associated with dysfunction in the DA pathway in AD using 4-month-old 5xFAD mice. These mice manifest a notable abundance of Aβ plaques, yet they have not displayed motor impairment. To trace the projection of DA neurons originating from the VTA, we administered AAV-pUbC-EGFP [65] into the VTA of 4-month-old WT and 5xFAD mice. Since accumulation of axonal swelling, a hallmark of axon injury, was found in the vicinity of Aβ plaques [66], we examined the morphology of DA neuronal axons. Viral EGFP-tagged VTA neuronal axons of 5xFAD mice exhibited prominent axon swelling near the cell body in the VTA (Fig. 5A–C). The STR is the principal target of nigrostriatal DA projection. Higher magnification imaging of the STR showed EGFP^+^ fibers with TH expression in WT mice, whereas signals of TH were significantly reduced in 5xFAD mice (Fig. 5 D and E). The axon terminals expressing EGFP that extended from the VTA were predominantly swollen in the STR region (Fig. 5D). 3D mapping of the midbrain using iACT revealed a significant decrease in TH neurons at both the VTA and SN regions of 5xFAD mice compared to WT mice. These results are consistent with the previous study [62] (Fig. 5 F and G). Neuronal degeneration may be preceded by neurite dystrophy, a condition that ultimately results in brain atrophy. Without significant regional brain loss, the 6-month-old 5xFAD mice exhibit the progression of the initial stage of TH^+^ neuronal loss.

Although Thy1-α-synuclein (αSyn) mice demonstrated the expression of αSyn throughout all brain regions [67], our data showed that YFP^+^ neurons were clearly distinguishable from TH neurons in Thy1-YFP;5xFAD mice (Fig. 5H). DA neurons displayed YFP-labeled synaptic puncta on their soma, which were produced by other Thy1-YFP neurons. This observation indicates Thy1-driven APP and PSEN transgenes are not expressed in TH^+^ neurons, revealing that axonopathy in DA neurons was induced by non-cell autonomous processes concomitant with amyloid pathology. The lysosomal protein Lamp1 has been found in dystrophic neurites [68]. TH immunoreactivity was observed in Lamp1^+^ axonal swellings in STR (5I and 5J). This observation indicates disruption of the autophagic pathway in DA neurons, reminiscent of what is seen in another AD mouse model [27] and in a Parkinson’s disease (PD) mouse model [69]. These results indicate the proximal axons of VTA neurons have a swelling phenotype, specifically in 5xFAD mice, which could be a structural mechanism for the loss of synapse function. Our findings align with a prior study that highlighted EEG modifications in 5xFAD mice, implicating changes in DA transmission [62]. In 5xFAD mice, where the APP transgene is expressed under the Thy1 promoter, the cell-autonomous neuronal pathology of pyramidal neurons can emerge by the ectopic APP accumulation in the axon. However, DA neurons were solely affected by extracellular Aβ not by ectopic intraneuronal APP aggregation, potentially leading to disruptions in the DA circuitry.

**Fig. 6:**
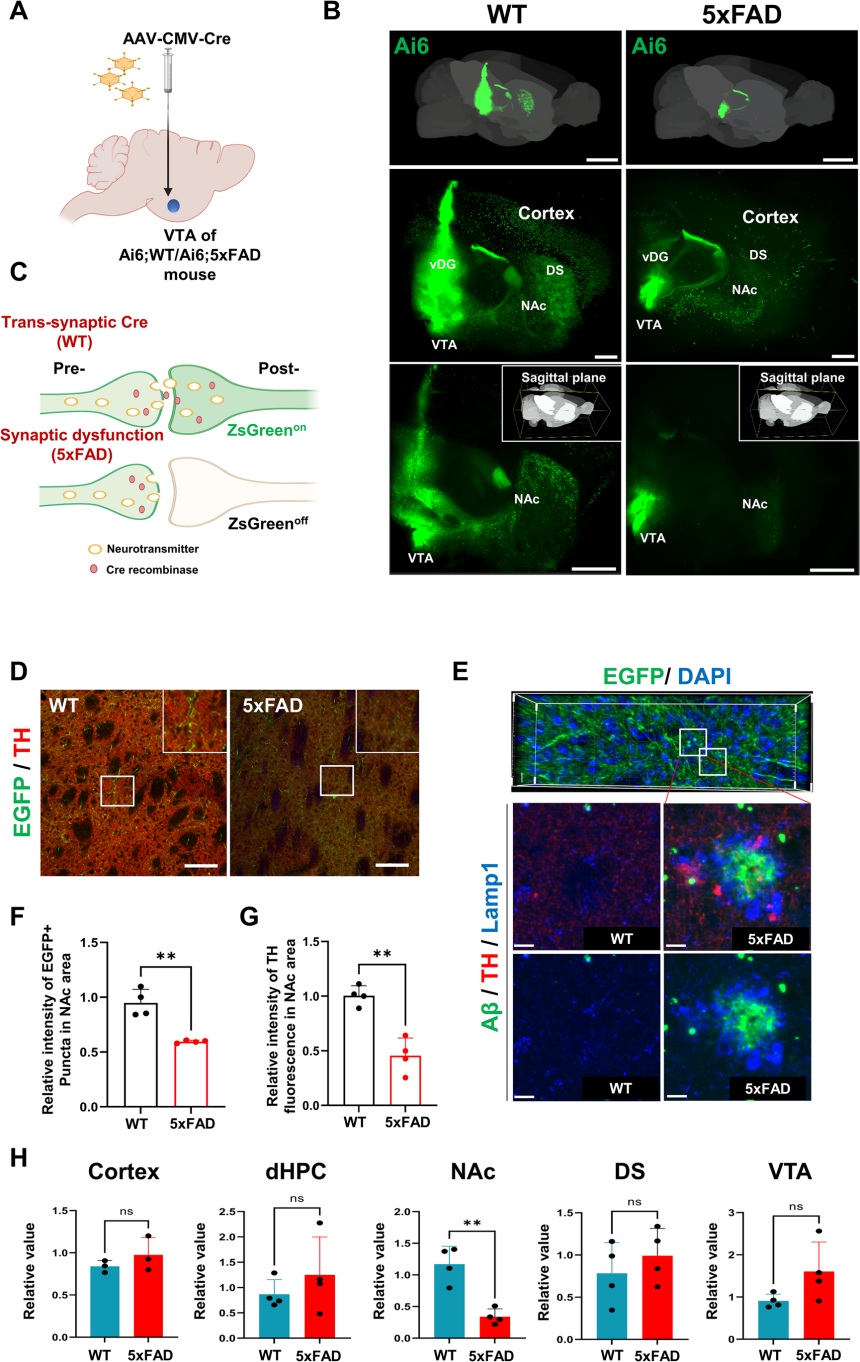
Swelling of DA neuronal axons reaching the NAc may underlie the DA release deficit. **A** Virus injection scheme. AAV carrying CMV-Cre was injected to the VTA of 4-month-old Ai6; WT or Ai6; 5xFAD mice. **B** Light sheet microscopy images of WT or Ai6; 5xFAD mouse brains in which AAV carrying CMV-GFP was injected into the VTA. Scale bars: 2000 μm. **C** A schematic diagram of synaptic transmission with AAV-Cre indicates synaptic inputs of VTA neurons were impaired in the 5xFAD mice. **D** Representative immunofluorescence images of coronal brain sections for TH in NAc of 4-month-old WT or 5xFAD mice that were injected with AAV carrying pUbC-EGFP into the VTA. Scale bars: 100 μm. **E** Representative immunofluorescence images for EGFP (upper image) and Aβ/TH/Lamp1 (bottom images) of coronal brain sections in the NAc of 4-month-old WT or 5xFAD mice that were injected with AAV carrying pUbC-EGFP into the VTA. Scale bars: 10 μm. **F** Relative intensity of EGFP signals in the VTA area of WT/5xFAD mice. **G** Measurement of the TH signal indicates abrogation of DA release from VTA to NAc in the 5xFAD mice. **H** DA levels were measured by HPLC in each region of the brain. dHPC, dorsal hippocampus; NAc, nucleus accumbens; DS, dorsal striatum; SN/VTA, substantia nigra/ventral tegmental area of 6-month-old 5xFAD mice

We further analyzed the synaptic transmissive ability of VTA neurons. AAV-carrying Cre recombinases (AAV-CMV-Cre) [70] were injected into the VTA of Ai6; WT and Ai6; 5xFAD mice (Fig. 6A). Transmission of Cre recombinases to target areas of VTA inputs was observed in Cre-recombined Ai6^+^ neurons in WT mice (Fig. 6B). AAV-Cre may be transferred to the next synaptically connected neuron, and the Cre reporter may visualize the AAV-Cre receiving neurons (Fig. 6C). Ai6^+^ neurons were mainly located in the VTA (the injected site of AAV-Cre), and Ai6 signals were also detected in the vDG, anterior cortex, dorsal striatum (DS), and NAc in WT mice (Fig. 6B), whereas Ai6^+^ neurons were limited in the VTA of 5xFAD mice, indicating impaired synaptic transmission of AAV-Cre from the VTA to target areas. The NAc is the principal target of mesolimbic DA projection. We investigated the projection of DA neurons from the VTA to the NAc in mice injected with AAV-pUbC-EGF (Fig. 5A). Higher magnification imaging of the NAc showed EGFP^+^ fibers with TH expression in WT mice, whereas signals of both EGFP and TH were significantly reduced in 5xFAD mice (Fig. 6D–G). The magnified images revealed axonal spheroids labeled with TH that coincided with Lamp1^+^ dystrophic neurites surrounding Aβ plaques. In order to explore the potential specificity of Aβ pathology in the mesolimbic DA pathway, we performed high-performance liquid chromatography coupled with electrochemical detection (HPLC-ECD) to measure DA levels in the cortex, HPC, NAc, DS, and VTA of 6-month-old WT and 5xFAD mice. A significant decrease in DA was found in the NAc, but not in other brain regions (Fig. 6H).

These results revealed that the mesolimbic pathway is preferentially damaged in 5xFAD mice compared to other DA-targeted regions, showing the possibility of compensatory mechanisms in response to the acute loss of DA neurons in VTA. This finding suggests that VTA axonal swelling in 5xFAD mice might be closely associated with the impairment of reciprocal neurotransmission between two regions, resulting in defective DA release in the NAc. The deficits in DA circuits could be responsible for the broad behavioral deficits in AD mice.

### DA Axonal Swelling and Mis-targeted Axons of DA Neurons Is Revealed by iACT

**Fig. 7:**
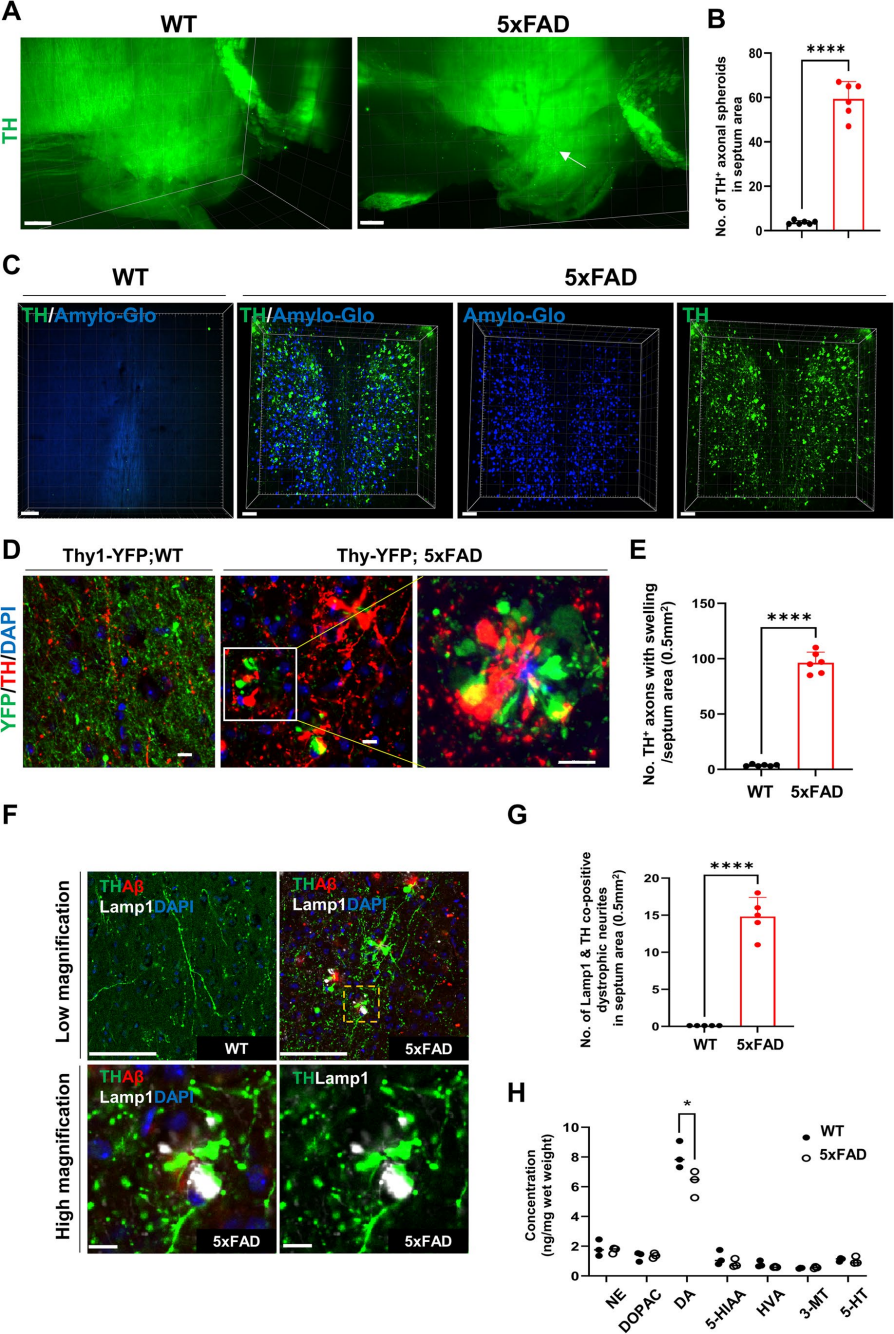
Mis-routed DA axons toward Aβ plaques and decreased DA release in the NAc. **A** 3D clearing tissue images revealed TH^+^ axonal aggregation in the septum region (white arrow) of the 5xFAD mice. Scale bars: 500 μm. **B** The number of axon swellings in the septum areas. **C** 3D imaging of 1-mm-thick slices of the septum area from WT or 5xFAD mice processed with the iACT protocol. Scale bars: 100 μm. **D** Representative images for Thy1-YFP and TH in the septum area of 4-month-old Thy1-YFP;WT or Thy1-YFP;5xFAD mice. Scale bars: 10 μm. **E** Quantification of the TH^+^ axonal swelling in the septum areas of WT/5xFAD mice. Scale bars, 10 μm. **F** Representative images for TH, Aβ, and Lamp1 in the septum area of WT/5xFAD mice. Scale bars: 100 μm (low magnification) and 10 μm (high magnification). **G** Quantification of TH and Lamp1 co-labeled dystrophic neurites in the septum area of WT/5xFAD mice. **H** NAc samples from 4-month-old WT or 5xFAD mice were analyzed by HPLC for norepinephrine (NE), 3,4-dihydroxyphenylacetic acid (DOPAC), DA, 5-hydroxyindoleacetic acid (5-HIAA), 5-hydroxytryptamine (5-HT), homovanillic acid (HVA), and 3-methoxytyramine

A few VTA DA neurons project to the septum [71]. Furthermore, we identified that a subset of TH-expressing neurons formed axonal spheroids that extended towards the septum (Fig. 7 A and B). The iACT method allowed clearer staining and visualization of aberrant projection of DA neuronal axons around Aβ plaques in the septum region from 5xFAD mice but not WT mice (Fig. 7C), thereby unveiling mis-targeted axons of DA neurons in AD. Likewise, observation of axonal swelling of TH neurons in the STR and NAc regions and immunolabeling of coronal brain sections from Thy1-YFP;5xFAD mice revealed that axons were swollen not only in YFP^+^ neurons but also in TH^+^ neurons simultaneously around the Aβ plaque in the septum (Fig. 7 D and E). TH^+^ axonal swellings were co-stained with anti-Lamp1 antibody, indicating the presence of neuronal dystrophy in DA neurons in septum (Fig. 7 F and G). These findings are reminiscent of amyloid-plaque-associated axonal spheroids, which disrupt axonal connectivity [72] due to toxic amyloid species that might cause impaired retrograde transport and the accumulation of endolysosomal vesicles in the axon terminal of DA neurons. The measurement of neurotransmitters in NAc lysates showed significantly reduced DA levels, whereas no significant differences were detected for norepinephrine (NE), 5-hydroxyindoleacetic acid (5-HIAA), 3-methoxytyramine (3-MT), and 5-hydroxytryptamine (5-HT) in 6-month-old 5xFAD mice (Fig. 7H). This might implicate abnormal trafficking of DA-containing synaptic vesicles to axonal targets via axon swelling and mis-routed axon terminals in the septum. Interestingly, amyloid pathology might affect DA metabolism, as evidenced by the unaltered levels of the DA metabolite, 3,4-dihydroxyphenylacetic acid (DOPAC), despite the reduction in DA level. Collectively, our data demonstrated that Aβ plaques caused synaptic dysfunction in DA neurons through phenotypic DA axonal alterations. Taken together, iACT as summarized in the graphical summary performed on 5xFAD mice, provided unforeseen structural neuropathy at the neural circuit level by visualizing the brain-wide DA pathways of 5xFAD mice and providing structural evidence for DA alterations.

## Discussion

Aβ is strongly associated with neuronal dysfunction and impaired synaptic plasticity. These deficits are accompanied by reduced hippocampal long-term potentiation (LTP) and enhanced long-term depression (LTD) [73, 74]. Furthermore, subcortical dysfunctions, including reduced levels of DA, have been observed in AD, indicating a correlation between psychiatric symptoms and cognitive decline [75]. Our advanced immunolabeling method for 3D optical imaging provides robust evidence that Aβ in 5xFAD mice disrupted DA circuits with axonal swelling of VTA-DA neurons and misrouted axon endings surrounding Aβ plaques.

Molecular labeling of brain-wide structures is required to understand the coordination between neural circuits and brain function. The ETC technique for lipid removal facilitates the molecular characterization of large-volume tissues using conventional immune-labeling methods [76]. However, the penetration of molecular labels into deep-tissue areas is challenging owing to the incomplete permeability of whole brain tissues [77]. Antibody penetration and binding of specific epitopes for fluorescent immunolabeling rely on a tissue permeabilization step using detergents or enzymes [78, 79]. Poor permeabilization can result in loss of cellular mass, including proteins [80] and tissue distortion [81]. Zwitterionic detergents form smaller micelles than other detergents such as Triton X-100 and Tween 20 and more rapidly move deeply into tissue, allowing the removal of excess cellular lipids in aged organs [82]. The zwitterionic detergent-based iACT method additionally enhanced antibody penetration through whole tissues. The zwitterionic detergent SB3-12 allowed molecular labels to penetrate deep tissue regions without substantial protein loss, as determined with LC-MS.

The PEGASOS method revealed that Quadrol decolorizes intact mouse brains. Quadrol combined with PEG solvents, achieved tissue transparency and preserved endogenous fluorescence for a long time [83]. A series of aminoalcohols, including Quadrol and triethanolamine, exhibited considerable tissue solubilizing activity [47]. We observed that Quadrol, a strong surfactant solubilizer, efficiently eliminated residual SDS after ETC-mediated brain clearing and led to high-throughput imaging of deep brain areas. This optimized method for the solubilization of large-volume tissues could vastly improve the 3D structural and molecular mapping of cells throughout the whole brain.

Liebmann et al. studied Aβ formation in an Alzheimer’s disease (AD) mouse model using iDISCO [84]. They demonstrated the 3D spatial distribution of Aβ plaques, microglia, and vasculatures. Optimized SWITCH whole-brain clearing and immunolabeling allowed a spatially unbiased map of the progression of Aβ deposition that displayed area-specific aggregation over time and revealed susceptible subcortical regions, such as the mammillary body in the 5xFAD mouse model [85]. Therefore, optical clearance combined with immunolabeling of large-volume tissues has the potential to unveil molecular pathology through deep fluorescence microscopic imaging.

In this study, we further determined the implication of amyloidopathy in alterations of the DA system using a rapid ETC-compatible clearing and labeling method. Lipids are essential for brain function, considering their roles in cellular composition, signaling, and energy metabolism. In the brain, cholesterol, hexosylceramide, and phosphatidylethanolamine are involved in axonal myelination and signal transduction [86, 87]. In AD, increased neuroinflammation has been linked to neural membrane damage and lipid modification [88]. These AD features of abnormal lipidomes restrict antibody accessibility to deep tissue areas [89]. We found that SB3-12 efficiently solubilized AD-specific lipid species, putatively determining eight kinds of lipid classes, including acyl carnitine, fatty acids, lysophosphatidylcholine, lysophosphatidylethanolamine, lysophosphatidylserine, NAE, NAT, and SPB, which led to clearer imaging of Aβ deposition in subcortical regions through improvement of antigen recovery in AD brain.

iACT exhibited reduced axonal projections of SN/VTA neurons into the NAc in 5xFAD mice. Despite whole brain imaging of 5xFAD mice showing that overall TH level in NAc was seen to be similar to that in WT mice, magnified analysis using 2D IHC and DA measurement revealed axonal swelling was locally associated with a deficit of DA release from VTA to NAc. Injection of AAV-CMV-Cre into the VTA of Ai6; 5xFAD mice successfully visualized the impaired neurotransmission of VTA-DA neurons to the NAc. Consistent with the stereotaxic experiments of AAV-Cre in Ai6 mice, the VTA of 5xFAD mice infused with viral particles carrying AAV-pUbC-EGFP, compared with WT mice, showed reduced GFP-expressing axons in projection targets such as NAc, vDG, and mPFC. Axon tracts with focal swelling form varicosities or larger spheroids in AD [90]. These abnormalities often occur without overt axonal disintegration; however, they likely contribute to significant functional impairments [3]. Increasing evidence suggests that the swelling of axons and apical dendrites is associated with intraneuronal Aβ accumulation in their cell bodies [91] and this can be a prerequisite for accelerated tau fibrillization. A previous study on axonopathy using whole-brain 3D profiling revealed numerous axonal spheroids in the cortical and subcortical regions of the brains of 5xFAD/Thy1-GFP mice [92]. Herein, we demonstrated that 3D optical imaging using iACT efficiently visualized the significant loss of mesolimbic DA projections. Furthermore, we observed swelling of the proximal regions of VTA-DA neuronal axons located around the Aβ plaque in the septum. These phenotypical axonal dystrophies are strongly linked to impaired DA transport from the VTA to the NAc in 5xFAD mice. Our study is limited to a mouse model, even though iACT aids in the understanding of subcortical Aβ pathology associated with DA circuitry. Further studies are, therefore, required to determine whether human AD brain tissues have pathologically identical phenotypes in the DA neuronal circuits. Our brain-wide analysis of the AD mouse brain using iACT supports a previous report using a Tg2576 AD mouse model in which a human APP-Swedish mutation led to a pre-plaque stage degeneration of DA neurons in the VTA region [28]. The loss of DA neurons in the VTA precedes the deposition of Aβ plaques in the HPC [93]. This indicates that soluble Aβ oligomers are capable of attacking DA neurons over long distances.

## Conclusions

Recent evidence has demonstrated correlations between subcortical and brainstem regions and neurodegenerative diseases, such as AD [94], PD [95], and amyotrophic lateral sclerosis [96]. Our newly developed rapid clearing and immunolabeling method was efficient in revealing the deep brain regions with volumetric imaging, and this method will facilitate the full characterization of the extent of neuropathology in deeper subcortical areas in future studies. Axonal dystrophy observed in brain-wide volumetric imaging could be an initial step of DA circuit-specific brain malfunctions in AD. Treating axonal dystrophy could be beneficial for the amelioration of multiple symptoms of AD.

### Supplementary information

ESM 1 (PDF 813 KB)

## Data Availability

Data are available online version of this manuscript.

## Abbreviations

iACT: Immuno-active clearing technique
SB3-12: N-Dodecyl-N,N-dimethyl-3-ammonio-1-propanesulfonate
Quadrol: 1% N,N,N′,N′-Tetrakis (2-hydroxypropyl)ethylenediamine
DA: Dopamine
CMC: Critical micelle concentration
Aβ: Amyloid-beta
AD: Alzheimer’s disease
HPC: Hippocampus
NAc: Nucleus accumbens
2D: Two-dimensional
ACT-PRESTO: Active clarity technique-pressure related efficient and stable transfer of macromolecules into organs
SDS: Sodium dodecyl sulfate
EEG: Electroencephalogram
ETC: Electrophoretic tissue clearing
VTA: Ventral tegmental area
TBST: Tris-buffered saline with 0.1% Tween™ 20
Ai6: ROSA26-loxP-STOP-loxP-ZsGreen
Ai9: ROSA26-loxP-STOP-loxP-tdTomato
PFA: Paraformaldehyde
RT: Room temperature
PBST: Phosphate Buffered Saline with 0.1% Tween™ 20
RI: Refractive index
PEI: Polyethylenimine
PEG: Polyethylene glycol
Avertin: 2,2,2-Tribromoethyl alcohol in tert-amyl alcohol
mPFC: Medial prefrontal cortex
vDG: Ventral dentate gyrus
BCA: Protein quantification using bicinchoninic acid
LC: Liquid chromatography
MS: Mass spectrometry
MALDI: Matrix-assisted laser desorption ionization
CHCA: α-Cyano-hydroxy-cinnamic acid
LMSD: LIPID MAPS® Structure Database
ROC: Receiver Operating characteristic
IHC: Immunohistochemistry
MBP: Myelin basic protein
LC-MS: Liquid chromatography-mass spectrometry
NF: Neurofilament
AAV: Adeno-associated virus
BNST: Bed nucleus of the stria terminalis
HY: Hypothalamus
TH: Tyrosine hydroxylase
STR: Striatum
BLA: Basolateral amygdala
AUC: Area under the curve
FAD: Familial AD
αSyn: α-Synuclein
PD: Parkinson’s disease
DS: Dorsal striatum
HPLC-ECD: High-performance liquid chromatography coupled with electrochemical detection
dDG: Dorsal dentate gyrus
NE: Norepinephrine
DOPAC: 3,4-Dihydroxyphenylacetic acid
5-HIAA: 5-Hydroxyindoleacetic acid
3-MT: 3-Methoxytyramine
5-HT: 5-Hydroxytryptamine
LTP: Long-term potentiation
LTD: Long-term depression
NAT: N-Acyl taurines
LPA: Lysophosphatidic acid
FOH: Fatty alcohols
WE: Wax esters
ST: Sterols
SPB: Sphingoid bases
SFE: Short fatty esters
NAE: N-Acyl enthanolamines
FA: Fatty acids
