# Correction: Advanced Immunolabeling Method for Optical Volumetric Imaging Reveals Dystrophic Neurites of Dopaminergic Neurons in Alzheimer’s Disease Mouse Brain

**DOI:** 10.1007/s12035-023-03878-8

**Published:** 2023-12-19

**Authors:** Soonbong Baek, Jaemyung Jang, Hyun Jin Jung, Hyeyoung Lee, Youngshik Choe

**Affiliations:** 1 Developmental Disorders & Rare Diseases Research Group, Korea Brain Research Institute, 61 Cheomdan-ro, Daegu, 41062 Republic of Korea; 2 Division of Applied Bioengineering, Dong-eui University, Busanjin-gu, Busan, 47340 Republic of Korea

**Correction** **:** **Molecular Neurobiology**

10.1007/s12035-023-03823-9

The original version of this article unfortunately contained mistake in one of the authors' name.

Specifically, the name "Soongbong Baek" should be replaced with "Soonbong Baek."

The original article has been corrected.

