# Supplementary material for: Advanced Immunolabeling Method for Optical Volumetric Imaging Reveals Dystrophic Neurites of Dopaminergic Neurons in Alzheimer’s Disease Mouse Brain
Source: Mol Neurobiol. 2023 Dec 4;61(7):3976–99. doi: 10.1007/s12035-023-03823-9 (PMC11236860; doi:10.1007/s12035-023-03823-9)
Supplement: Supplementary file 1 — ESM 1 (PDF 813 KB) [file 12035_2023_3823_MOESM1_ESM.pdf]

Advanced immunolabeling method for optical volumetric imaging reveals dystrophic neurites of dopaminergic neurons in Alzheimer's disease mouse brain

Short running title: Visualization of dopaminergic axonopathy

Soongbong Baek[#,1], Jaemyung Jang[#,1], Hyun Jin Jung[1], Hyeyoung Lee[2], and Youngshik Choe[1*]

Supplementary Figures

Supplementary Figure 1. (A) Representative images of Tuj1$^+$ neurites in cortical tissues from 4-month-old mice. IHC was performed after incubation of the brain slice with PBST, 4 % SDS, and 4 % SB3-12 at RT for 24 hr. Scale bar: 10 µm. (B) Tuj1$^+$ neurites at 50 µm z-depth were quantified. In each condition, images (size 184 µm × 184 µm,) were acquired on random fields. (C) Quantification of Tuj1 fluorescence signal at 50 µm z-depth. In each condition, images (size 184 µm × 184 µm,) were acquired on random fields.

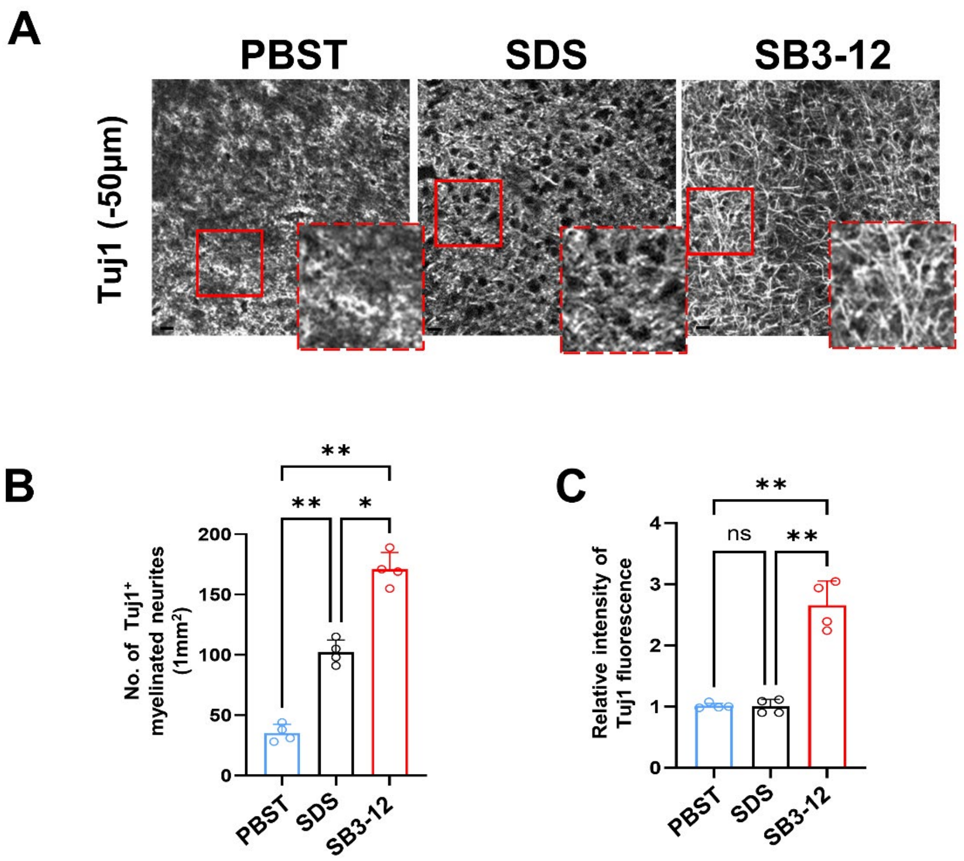

Supplementary Figure 2. (A) Immunofluorescence images of the hemisphere of 4-month-old Thy-YFP mice performed by iACT. (B) Light sheet microscopy images demonstrate the Nkx2.1-Cre-induced ZsGreen Cre reporter and anti-TH fluorescence of the Nkx2.1-Cre; Ai6 (ROSA26-loxP-STOP-loxP-ZsGreen) mouse hemisphere. OB: olfactory bulb, VTA: ventral tegmental area, HY: hypothalamus, BNST: the bed nucleus of the stria terminalis. (C) Comparison of ACT without immunostaining and iACT procedures using anti-GFP antibodies. Brain tissues were obtained from 4-month-old C57BL/6 mice injected with AAV2.retro-EGFP in vDG. vDG: ventral dentate gyrus. (D) iACT with anti-GFP antibody visualized GFP labeled axons of 4-month-old C57BL/6 mouse brains that were injected with AAV carrying GFP into the mPFC. mPFC: medial prefrontal cortex, BLA: Basolateral amygdala. NAc: nucleus accumbens. VTA: ventral tegmental area.

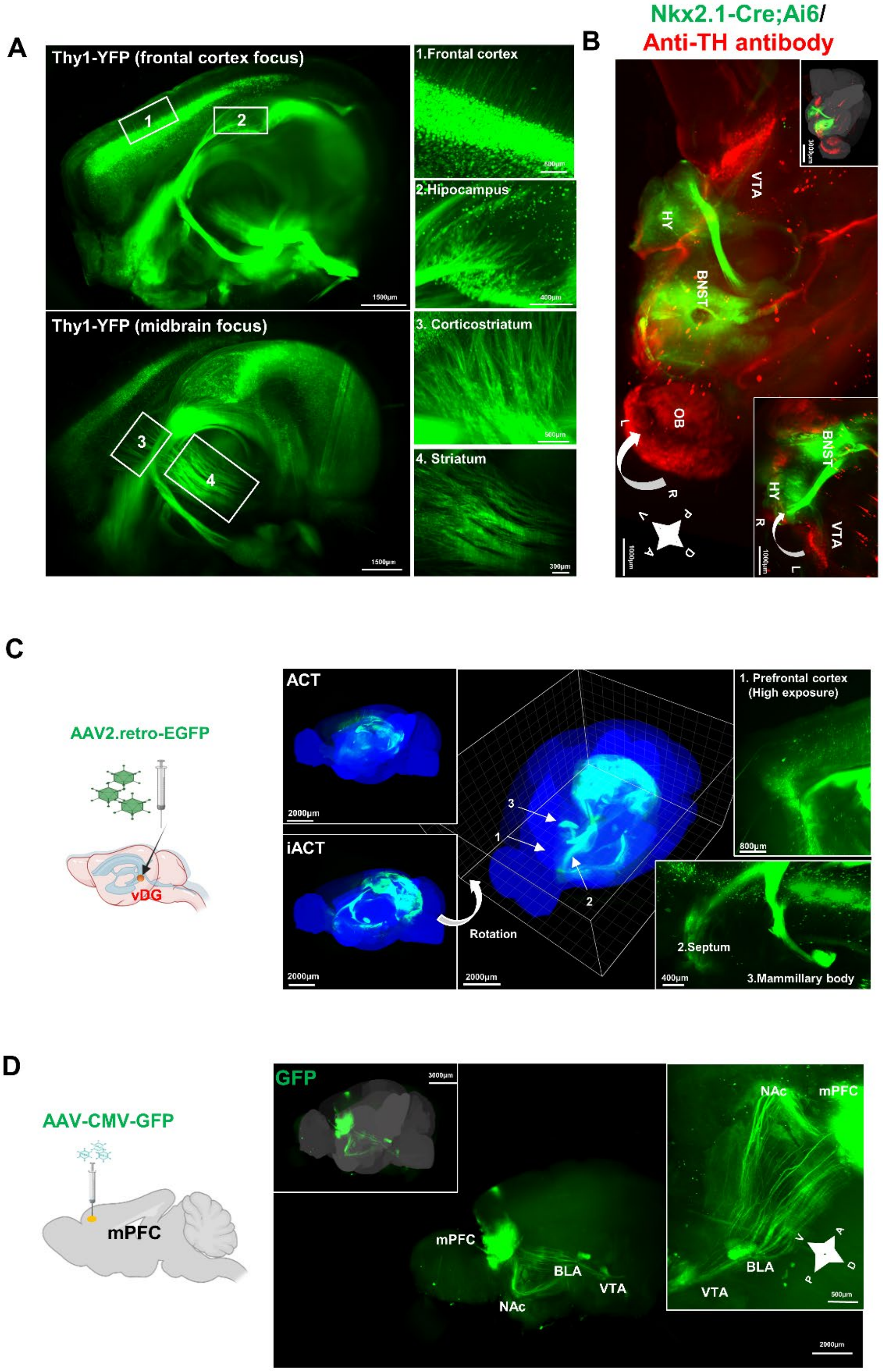
A
Thy1-YFP (frontal cortex focus)
1.Frontal cortex
2.Hipocampus
Thy1-YFP (midbrain focus)
3. Corticostriatum
4. Striatum
1500μm
B
Nkx2.1-Cre;Ai6/
Anti-TH antibody
VTA
HY
BNST
OB
1000μm
C
AAV2.retro-EGFP
vDG
ACT
iACT
Rotation
2000μm
1. Prefrontal cortex
(High exposure)
800μm
2.Septum
3.Mammillary body
400μm
D
AAV-CMV-GFP
mPFC
GFP
3000μm
mPFC
BLA
VTA
NAc
mPFC
500μm
2000μm

Supplementary Figure 3. (A) PS19; Thy-YFP brain tissues stained with antibody against GFP following iACT procedure. Non-immunolabeling control was performed following ACT procedure. White arrows indicate dystrophic neurons.

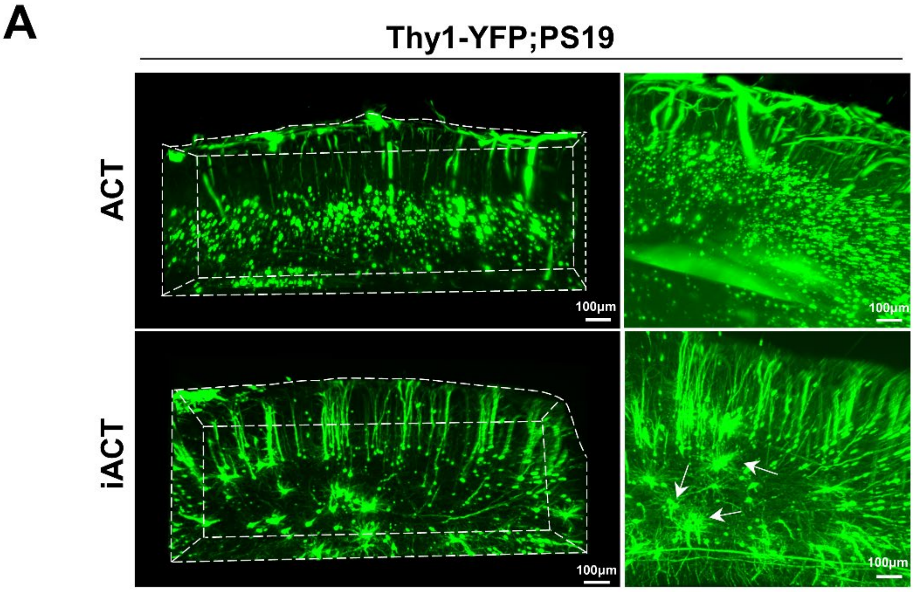

Supplementary Table 1. List of differentially expressed proteins from the Quadrol-washed samples by comparing the PBS-washed samples. Differential protein expression analysis was performed with Proteome Discoverer 2.4 (Thermo Scientific)

Supp. Table 1 is supplied as a csv file.

Supplementary Table 2. List of 90 LIPID MAPS database entries used as source database for mining the 9 identified precursor peaks derived from MALDI-MS analysis on WT mouse brain tissues permeabilized by SB3-12.

matched_mass	m/z	delta	lipids	ion	type
358.1754	358.1553	.0201	SPBP 15:2;O2	[M+Na]+	Sphingolipids
358.1989	358.1553	.0436	SPBP 14:0;O4	[M+H]+	Sphingolipids
358.1990	358.1553	.0437	NAE 14:0;O3	[M+K]+	Fatty acyls
358.2013	358.1553	.0460	ST 19:4;O3;G	[M+H]+	Sterol lipids
358.2022	358.1553	.0469	NAT 14:0	[M+Na]+	Fatty acyls
358.2047	358.1553	.0493	NAT 16:3	[M+H]+	Fatty acyls
267.1238	267.0903	.0335	FA 14:4;O3	[M-H]-	Fatty acyls
267.1369	267.0903	.0466	FA 12:0;O2	[M+Cl]-	Fatty acyls
267.1391	267.0903	.0488	ST 18:5;O2	[M-H]-	Sterol lipids
450.1665	450.1813	.0148	LPS O-10:0;O	[M+Cl]-	Glycerophospholipids
450.1689	450.1813	.0124	ST 21:5;O5;G	[M+Cl]-	Sterol lipids
450.1723	450.1813	.0090	ST 18:1;O4;T	[M+Cl]-	Sterol lipids
450.1723	450.1813	.0090	NAT 18:4;O2	[M+Cl]-	Fatty acyls
450.1770	450.1813	.0043	ST 20:4;O8;G	[M-H]-	Sterol lipids
450.1899	450.1813	.0086	LPS O-13:3;O	[M-H]-	Glycerophospholipids
450.1899	450.1813	.0086	LPS 13:2	[M-H]-	Glycerophospholipids
450.1900	450.1813	.0087	CAR 13:3;O4	[M+Cl]-	Fatty acyls
450.1900	450.1813	.0087	ST 18:0;O7;G	[M+Cl]-	Sterol lipids
450.1956	450.1813	.0143	ST 21:4;O4;T	[M-H]-	Sterol lipids
450.2053	450.1813	.0240	ST 22:4;O4;G	[M+Cl]-	Sterol lipids
450.2087	450.1813	.0274	ST 19:0;O3;T	[M+Cl]-	Sterol lipids
450.2087	450.1813	.0274	NAT 19:3;O	[M+Cl]-	Fatty acyls
450.2133	450.1813	.0321	ST 21:3;O7;G	[M-H]-	Sterol lipids
450.2167	450.1813	.0354	NAT 18:2;O4	[M-H]-	Fatty acyls

matched_mass	m/z	delta	lipids	ion	type
450.2262	450.1813	.0450	LPE 15:2;O	[M-H]-	Glycerophospholipids
450.2262	450.1813	.0450	LPS O-14:2	[M-H]-	Glycerophospholipids
450.2262	450.1813	.0450	LPC 12:2;O	[M-H]-	Glycerophospholipids
450.2264	450.1813	.0451	CAR 14:2;O3	[M+Cl]-	Fatty acyls
450.2286	450.1813	.0473	ST 25:7;O4;G	[M-H]-	Sterol lipids

Supplementary Table 3. Matching of mass spectra to LIPID MAPS database from MALDI-MS analysis of 5xFAD mouse brain tissues permeabilized by SB3-12. The positive peak of m/z 358. 1553 was exclusively derived from SB3-12-washed 5x FAD mouse brain, whereas the negative peak of m/z 450.1833 was obtained from SB3-12-washed and SDS-washed 5xFAD brain. The second peak of m/z 267.0903 was obtained from comparisons between SB3-12-washed and PBS-washed 5xFAD brain tissues.

matched_mass	m/z	delta	lipids	ion	type
359.1020	359.1243	.0222	LPA O-11:3	[M+K]+	Glycerophospholipids
359.1230	359.1243	.0013	LPA O-11:3;O	[M+Na]+	Glycerophospholipids
359.1230	359.1243	.0013	LPA 11:2	[M+Na]+	Glycerophospholipids
359.1255	359.1243	.0013	ST 18:3;O5	[M+K]+	Sterol lipids
359.1287	359.1243	.0045	ST 18:3;O;S	[M+Na]+	Sterol lipids
359.1465	359.1243	.0222	ST 18:3;O6	[M+Na]+	Sterol lipids
359.1594	359.1243	.0351	LPA O-12:2	[M+Na]+	Glycerophospholipids
359.1619	359.1243	.0376	FA 19:5;O2	[M+K]+	Fatty acyls
359.1619	359.1243	.0376	ST 19:2;O4	[M+K]+	Sterol lipids
292.2049	292.2508	.0459	SPB 15:1;O2	[M+Cl]-	Sphingolipids
292.2049	292.2508	.0459	NAE 13:0	[M+Cl]-	Fatty acyls
358.0733	358.0723	.0010	NAT 10:2;O3	[M+Cl]-	Fatty acyls
358.1097	358.0723	.0374	NAT 11:1;O2	[M+Cl]-	Fatty acyls
361.1115	361.1057	.0058	ST 19:5;O2;S	[M-H]-	Sterol lipids
361.1189	361.1057	.0132	LPA O-10:1;O	[M+Cl]-	Glycerophospholipids
361.1189	361.1057	.0132	LPA 10:0	[M+Cl]-	Glycerophospholipids
361.1293	361.1057	.0236	ST 19:5;O7	[M-H]-	Sterol lipids
361.1422	361.1057	.0365	LPA 13:3	[M-H]-	Glycerophospholipids
361.1423	361.1057	.0367	FA 17:4;O4	[M+Cl]-	Fatty acyls
361.1479	361.1057	.0422	ST 20:4;O;S	[M-H]-	Sterol lipids
361.1552	361.1057	.0495	LPA O-11:0	[M+Cl]-	Glycerophospholipids

matched_mass	m/z	delta	lipids	ion	type
363.1578	363.1244	.0334	LPA O-13:3;O	[M-H]-	Glycerophospholipids
363.1578	363.1244	.0334	LPA 13:2	[M-H]-	Glycerophospholipids
371.1631	371.2095	.0464	FA 19:5;O3	[M+Cl]-	Fatty acyls
371.1631	371.2095	.0464	ST 19:2;O5	[M+Cl]-	Sterol lipids
371.2592	371.2095	.0497	FA 24:6;O	[M-H]-	Fatty acyls
371.2592	371.2095	.0497	ST 24:3;O3	[M-H]-	Sterol lipids
450.1535	450.1115	.0420	LPS 12:3;O	[M-H]-	Glycerophospholipids
450.1592	450.1115	.0477	ST 20:5;O5;T	[M-H]-	Sterol lipids
453.1144	453.1643	.0498	ST 22:6;O3;S	[M+Cl]-	Sterol lipids
453.1225	453.1643	.0418	ST 21:5;O6;S	[M-H]-	Sterol lipids
453.1322	453.1643	.0321	ST 22:6;O8	[M+Cl]-	Sterol lipids
453.1356	453.1643	.0287	ST 19:1;O5;S	[M+Cl]-	Sterol lipids
453.1436	453.1643	.0206	ST 18:0;O8;S	[M-H]-	Sterol lipids
453.1451	453.1643	.0192	LPA 16:4;O	[M+Cl]-	Glycerophospholipids
453.1508	453.1643	.0135	ST 23:5;O2;S	[M+Cl]-	Sterol lipids
453.1531	453.1643	.0111	BMP 12:2;O	[M-H]-	Glycerophospholipids
453.1589	453.1643	.0054	ST 22:4;O5;S	[M-H]-	Sterol lipids
453.1686	453.1643	.0043	ST 23:5;O7	[M+Cl]-	Sterol lipids
453.1719	453.1643	.0077	ST 20:0;O4;S	[M+Cl]-	Sterol lipids
453.1815	453.1643	.0172	LPA 17:3	[M+Cl]-	Glycerophospholipids
453.1815	453.1643	.0172	LPA O-17:4;O	[M+Cl]-	Glycerophospholipids
453.1872	453.1643	.0229	ST 24:4;O;S	[M+Cl]-	Sterol lipids
453.1895	453.1643	.0253	LPG 13:2;O	[M-H]-	Glycerophospholipids
453.1895	453.1643	.0253	BMP 13:1	[M-H]-	Glycerophospholipids
453.1953	453.1643	.0310	ST 23:3;O4;S	[M-H]-	Sterol lipids
453.2049	453.1643	.0407	ST 18:3;O;Hex	[M+Cl]-	Sterol lipids
453.2049	453.1643	.0407	DG 21:6;O	[M+Cl]-	Glycerolipids
453.2049	453.1643	.0407	ST 24:4;O6	[M+Cl]-	Sterol lipids

matched_mass	m/z	delta	lipids	ion	type
453.2105	453.1643	.0462	ST 27:7;O;S	[M-H]-	Sterol lipids
459.2153	459.2642	.0489	LPA 19:4;O	[M-H]-	Glycerophospholipids
459.2155	459.2642	.0487	DG 20:4;O2	[M+Cl]-	Glycerolipids
459.2155	459.2642	.0487	ST 23:2;O7	[M+Cl]-	Sterol lipids
459.2211	459.2642	.0432	ST 26:5;O2;S	[M-H]-	Sterol lipids
459.2284	459.2642	.0358	LPA 17:0	[M+Cl]-	Glycerophospholipids
459.2284	459.2642	.0358	LPA O-17:1;O	[M+Cl]-	Glycerophospholipids
459.2308	459.2642	.0335	ST 27:6;O4	[M+Cl]-	Sterol lipids
459.2341	459.2642	.0301	ST 24:1;O;S	[M+Cl]-	Sterol lipids
459.2388	459.2642	.0254	ST 26:5;O7	[M-H]-	Sterol lipids
459.2388	459.2642	.0254	ST 20:3;O;GlcA	[M-H]-	Sterol lipids
459.2388	459.2642	.0254	ST 20:4;O2;Hex	[M-H]-	Sterol lipids
459.2422	459.2642	.0220	ST 23:0;O4;S	[M-H]-	Sterol lipids
459.2517	459.2642	.0125	PA O-20:3	[M-H]-	Glycerophospholipids
459.2517	459.2642	.0125	LPA O-20:4;O	[M-H]-	Glycerophospholipids
459.2517	459.2642	.0125	LPA 20:3	[M-H]-	Glycerophospholipids
459.2519	459.2642	.0123	DG 21:3;O	[M+Cl]-	Glycerolipids
459.2519	459.2642	.0123	ST 18:0;O;Hex	[M+Cl]-	Sterol lipids
459.2519	459.2642	.0123	FA 24:4;O4	[M+Cl]-	Fatty acyls
459.2519	459.2642	.0123	DG O-21:4;O2	[M+Cl]-	Glycerolipids
459.2519	459.2642	.0123	ST 24:1;O6	[M+Cl]-	Sterol lipids
459.2575	459.2642	.0068	ST 27:4;O;S	[M-H]-	Sterol lipids
459.2648	459.2642	.0006	LPA O-18:0	[M+Cl]-	Glycerophospholipids
459.2672	459.2642	.0029	ST 28:5;O3	[M+Cl]-	Sterol lipids
459.2752	459.2642	.0110	ST 27:4;O6	[M-H]-	Sterol lipids
459.2752	459.2642	.0110	ST 21:3;O;Hex	[M-H]-	Sterol lipids
459.2752	459.2642	.0110	FA 27:7;O4	[M-H]-	Fatty acyls
459.2752	459.2642	.0110	DG 24:6;O	[M-H]-	Glycerolipids

matched_mass	m/z	delta	lipids	ion	type
459.2881	459.2642	.0239	LPA O-21:3	[M-H]-	Glycerophospholipids
459.2883	459.2642	.0240	DG 22:2	[M+Cl]-	Glycerolipids
459.2883	459.2642	.0240	MG 22:3;O	[M+Cl]-	Glycerolipids
459.2883	459.2642	.0240	FA 25:3;O3	[M+Cl]-	Fatty acyls
459.2883	459.2642	.0240	DG O-22:3;O	[M+Cl]-	Glycerolipids
459.2883	459.2642	.0240	ST 25:0;O5	[M+Cl]-	Sterol lipids
459.3035	459.2642	.0393	ST 29:4;O2	[M+Cl]-	Sterol lipids
459.3035	459.2642	.0393	FA 29:7	[M+Cl]-	Fatty acyls
459.3116	459.2642	.0474	FA 28:6;O3	[M-H]-	Fatty acyls
459.3116	459.2642	.0474	DG O-25:6;O	[M-H]-	Glycerolipids
459.3116	459.2642	.0474	MG 25:6;O	[M-H]-	Glycerolipids
459.3116	459.2642	.0474	DG 25:5	[M-H]-	Glycerolipids
459.3116	459.2642	.0474	ST 28:3;O5	[M-H]-	Sterol lipids

Supplementary Table 4. List of antibodies used for immunofluorescence studies.

Primary antibody	Manufacture (Cat. No.)	Clone	Dilution
Purified anti-Tubulin β 3 (TUBB3) antibody	BioLegend (801201)	Mouse $IgG2_{a, k}$	1:200
Anti-Myelin Basic Protein antibody	Aves Labs (MBP)	Chicken IgY	1:100
GFP (B-2) Alexa Fluor 488 antibody	Santa Cruz (sc9996)	Mouse $IgG2_{a}$	1:200
GFP DyLight 488 antibody	Novus (NBP1-69963)	Mouse IgG	1:200
Anti-NeuN antibody, clone A60	Millipore (MAB377)	Mouse IgG	1:100
Purified anti-Neurofilament Marker (pan axonal, cocktail) antibody	BioLegend (837902)	Mouse $IgG1_{, k}$/IgM,	1:100
Anti-Tyrosine Hydroxylase antibody	Pel-Freez (P40101-150)	Rabbit polyclonal	1:100
Tyrosine Hydroxylase antibody	Immunostar (22941)	Mouse IgG1	1:100
Anti-Collagen IV antibody	Abcam (ab6586)	Rabbit polyclonal	1:200
β-Amyloid XP® antibody, clone D54D2	Cell Signaling Technology (8243S)	Rabbit monoclonal	1:200
1D4B (Lamp1) antibody	DSHB (1D4b)	Rat IgG2a	1:200
